# A Robust Atomically Precise Nanocluster Catalyst for Simultaneous C–O and C–C Bond Cleavage in Lignin Models

**DOI:** 10.1021/acscatal.5c07479

**Published:** 2025-11-24

**Authors:** Zhaoxian Qin, Akanksha Lakra, Rahul R Somni, Wenbo Peng, Gao Li, Guoxiang Hu, Zhaohui Tong

**Affiliations:** a School of Chemical and Biomolecular Engineering, 1372 Georgia Institute of Technology, Atlanta, Georgia 30332, United States; b School of Chemistry and Chemical Engineering, 71203 Inner Mongolia Normal University, Hohhot 010018, China; c School of Materials Science and Engineering, 1372 Georgia Institute of Technology, Atlanta, Georgia 30332, United States

**Keywords:** gold nanoclusters, nanocatalyst, lignin depolymerization, biomass conversion, C−C bond cleavage

## Abstract

The emerging metal nanoclusters are ideal molecular models for the catalysts in terms of their atomically precise structures and structure-tailored catalytic performance. However, designing an effective and robust nanocluster-based catalyst is challenging owing to its complicated synthesis approaches, poor stability, and unknown reaction mechanism. Herein, a body-centered cubic gold nanocluster, [Au_9_(Dppy)_8_]^3+^, is reported, which exhibits high stability in CH_2_Cl_2_ compared with other Au_9_ clusters owing to its chemical charge and tight monomolecular protection layer. On TiO_2_ nanoparticles, this Au_9_ nanocluster could keep a single-cluster status to give a nanocatalyst that has strong chemisorption to H_2_ and survives under harsh reaction conditions due to the strong metal–supporter interaction (SMSI). The TiO_2_-supported Au_9_ has served as an effective catalyst to cleave the C–C and C–O bonds of lignin model compounds, 1-(3,4-methoxyphenyl)-2-(2-methoxyphenyl)­propane-1,3-diol (LD), achieving an impressive conversion rate (92%). Moreover, the activity of nanocluster-based catalysts could vary according to the structures of nanoclusters loaded in comparison, demonstrating that the catalytic performance of nanocluster-based catalysts can be tailored by regulating nanoclusters’ structures, including the core sizes, shapes, surface ligands, and metal chemical status. Finally, the durability of this nanocatalyst can be significantly improved using a ligand-free Au_9_/TiO_2_ catalyst, accompanied by a higher activity to C_α_–C_β_ cleavage and slightly reduced LD conversion yield, for which the distance between reactants and metal core should be responsible according to the DFT simulations.

## Graphical abstract

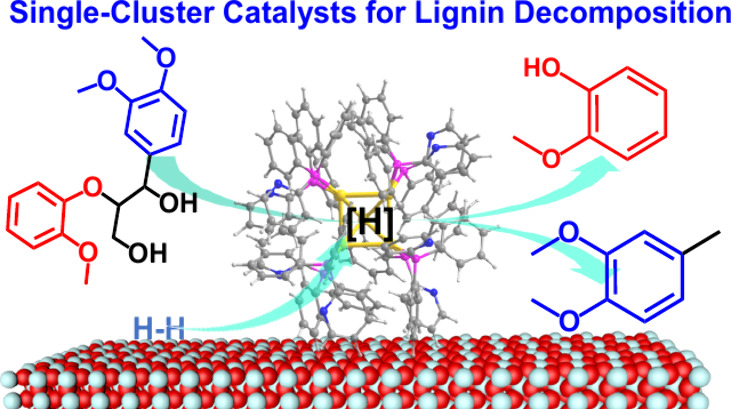

Lignin is the primary component in constructing plant cell walls and the only natural source of aromatic materials, due to its unique phenolic structures.^1–4^ This intricate structure makes lignin an attractive resource for conversion into biofuels and other value-added biochemicals like aromatics. According to the report from Grand View Research (GVR),^5^ the global lignin market was valued at approximately USD 1.08 billion in 2023, and it keeps increasing with an expected compound annual growth rate (CAGR) of 4.5% from 2024 to 2030. Despite its high production, lignin is largely underutilized as low-cost energy through incineration, and only 2% of lignin has been utilized as a value-added chemical or material.^5^ Lignin is a complex three-dimensional polymer composed of methoxylated phenylpropanoid units of various types linked by primarily C–O or C–C bonds.^6–9^ However, the depolymerization of lignin remains a formidable challenge due to its intrinsic network structure and the stability of its interlinking ether and C–C bonds, especially the high-energy C–C bonds.

Various strategies, including enzymolysis, pyrolysis, hydrolysis, reduction, oxidation, photocatalysis, electrocatalysis, and supercritical ethanol extraction, have been proposed to convert lignin into value-added chemicals using a series of catalyst systems, including various complexes, nonmetals (GO, acids, N_3_N_4_, etc.), carbon, or metal oxide-supported metals (Pt, Pd, Cu, W, Ru, Rh, *etc.*).^6,10–19^ For example, Liu and Dyson used oxidative cross-coupling reactions in the presence of Cu­(OTf)_2_ to extract the aromatic monomers from lignin and produce a functionalized diaryl ethers by breaking β–O–4 linkages, overcoming the limitations of oxidative methods.^18^ Stone et al. developed a continuous hydrodeoxygenation process based on the Mo_2_C catalyst,^15^ to successfully convert poplar lignin into aromatic hydrocarbons at 86% of the theoretical carbon recovery and with 87.5% selectivity toward aromatic hydrocarbons. Tong’s group developed a strategy to convert lignin into pure guaiacol, vanillin aldehyde (acid), and corresponding derivatives using the active oxygen species in graphene oxide (GO) as facile nanoscissors to efficiently cleave ether and C–C bonds.^14^ Dufour and co-workers studied the effects of lignin structures and carbon-supported metal catalysts (Pt/C, Ni/C, and Ru/C) in supercritical ethanol.^16^ These studies revealed that catalysts play a significant role in their conversion to monomers, oligomers, and chars by promoting cracking reactions and stabilizing the broken bonds via H-transfers. In addition, photocatalysts like Zn_2_In_2_S_4_, CdS/TiO_2_, and other chalcogenides have been used for photocatalytic self-transfer hydrogenolysis of lignin models and diverse lignin substrates.^10,17,20–22^ These studies mainly focus on the effects of catalysts on the performance of the lignin degradation processes. However, the impact of the catalyst structural design and size tuning of the catalyst on lignin interlinking bond cleavage is significant yet rarely discussed. In the emerging nanocatalyst area, it has been well recognized that the size of active sites in catalysts, such as single atoms, nanoclusters (<3 nm), and nanoparticles (3–100 nm), plays a significant role in their catalytic performance for many reactions.^23–26^ Recently, pioneer studies confirmed the difference in the catalytic performance of single atoms and nanoparticle catalysts in lignin depolymerization.^6,27–29^ Compared with single atoms and nanoparticles, nanoclusters, constructed by a few hundred metal atoms, have attracted much interest as catalysts due to their atomical and tunable nanostructures. Their unique nanostructure makes them an excellent catalyst candidate for fundamentally understanding how catalyst design and structures influence catalytical reaction performance. Until now, research has been largely limited due to the complicated preparation process and poor stability of nanoclusters.

In contrast with the unprotected metal nanoclusters,^30–33^ which consist solely of metal atoms, ligand-protected metal nanoclusters (LPMNCs)^34,35^ offer high uniformity in size distribution, chemical compositions, and crystal structures, along with enhanced flexibility for postsynthesis applications. Surface ligands on LPMNCs reduced the reliance on high-energy equipment required by unprotected nanoclusters and greatly enhanced the dispersion capability and stability of nanoclusters in most organic solvents. Thus, LPMNCs have been regarded as one of the most unique and promising nanomaterials in both fundamental structure–property studies and industrial applications, including photoluminescence, catalyst, medicine, bioimaging, biolabeling, sensing, nanodevices, and 3D printing,^35–43^ owing to their unique optical, magnetic, and electronic properties.^44–48^ Their subnanometer size distribution (<3 nm) offers them super high surface Gibb’s free energy and adsorption capability to small molecules (O_2_, H_2_, CO, CO_2_, etc.), exhibiting excellent catalytic performance in various reaction processes, such as oxidation, reduction, and coupling.^37,38,49–51^ Moreover, their catalytic performance is closely related to their atomical structures (ligands, metal atoms, metal core size, and metal–ligand interface), making them ideal molecular models for the catalysts.^52^ Despite the innate advantages of metal nanoclusters in catalysis, the challenges also exist. LPMNCs are hard to prepare on a large scale. Their characterization requires expensive and complicated instruments. Maintaining the nanoclusters’ characteristics, such as ultrasmall size, core structure, and high activity, in nanocatalysts is challenging during the catalyst preparation process and under harsh reaction conditions. These factors seriously hinder their employment in most reactions, including the depolymerization of lignin. These knowledge gaps have sparked our interest in exploring the potential of metal nanoclusters and subnanogold for upgrading lignin into value-added chemicals.

Herein, we present a straightforward, scalable nanocluster synthesis strategy for developing robust nanocluster-based catalysts with exceptional durability and catalytic performance. Through this strategy, a robust, six-electron metal, and body-centered nanocluster, [Au_9_(Dppy)_8_]^3+^ (abbreviated as Au_9_ hereafter, Dppy = 2-(diphenylphosphino)­pyridine), was synthesized in a simplified approach, achieving an exceptionally high yield of approximately 84%. Their properties were characterized by various advanced technologies, including UV–vis, electrospray ionization mass spectroscopy (ESI-MS), single-crystal X-ray diffraction (SCXRD), powder X-ray diffraction (PXRD), X-ray photoelectron spectroscopy (XPS), and nuclear magnetic resonance (NMR), to elucidate its formation, crystal structure, optical properties, and stability. Subsequently, a nanosized TiO_2_-supported Au_9_ catalyst (merely 1 wt %) was prepared successfully, and its catalytic performance was evaluated and compared with other TiO_2_-supported Au nanoclusters and Au nanoparticles in cleaving the C–O and C–C bonds of a lignin dimer model compound. The catalytic results show that our newly developed cubic Au_9_ with bridged surface ligands exhibits the best catalytic activity compared with other nanoclusters with different core shapes, sizes, and compositions. Furthermore, the surface ligands of nanoclusters were found to enhance catalyst activity, as evidenced by comparison with the TiO_2_-supported Au_9_ cluster without surface ligands. It is worth noting that bare Au_9_ clusters on TiO_2_ can achieve excellent catalyst durability and higher activity to C–C bond cleavage, although their activity is slightly reduced, for which the SMSI and interactions between gold core and reactants should be responsible. This work establishes a strategic framework for designing robust, highly active nanocluster-based catalysts and offers valuable insights into developing novel and effective nanocluster catalysts for cleaving C–C and C–O bonds in lignin.

## Results and Discussion

### Synthesis and Structure Determination

The cubic Au_9_ cluster was synthesized via the reduction of gold compounds, following a method described in our previous work.^53^ Briefly, a clear solution from the reaction of DppyAuCl (Dppy = diphenylphosphine pyridine) with AgSbF_6_ was slowly reduced by NaBH_4_ to yield the desired nanocluster (see more details in the Supporting Information). Here, the removal of Cl from the reaction system depressed the effects of counterions on the formation of gold nanoclusters since the Cl ion has a stronger coordination capability than that of SbF_6_^–^. Moreover, the formation of the roust cubic Au_9_ cluster depends on the introduced Dppy ligands since other clusters, like crown and butterfly like Au_9_ clusters, could be achieved if the Dppy ligand was replaced by only Ph_3_P, according to our previous work.^53^ Unlike other metal nanoclusters typically obtained in postreaction solutions, the Au_9_ cluster precipitated as a high-purity green solid at the bottom of the reactor (Figure 1a), simplifying both the isolation and purification processes. The *in situ* isolation of products from the reaction system, which is closely related to the solubility of the obtained Au_9_ nanoclusters in methanol and dichloromethane, is vital for the superhigh yield of 84% of the target product. Moreover, this phase separation also makes it possible to scale up the production of nanoclusters with high purity and high yield, which is hard to achieve for most metal nanoclusters.

**Figure 1:**
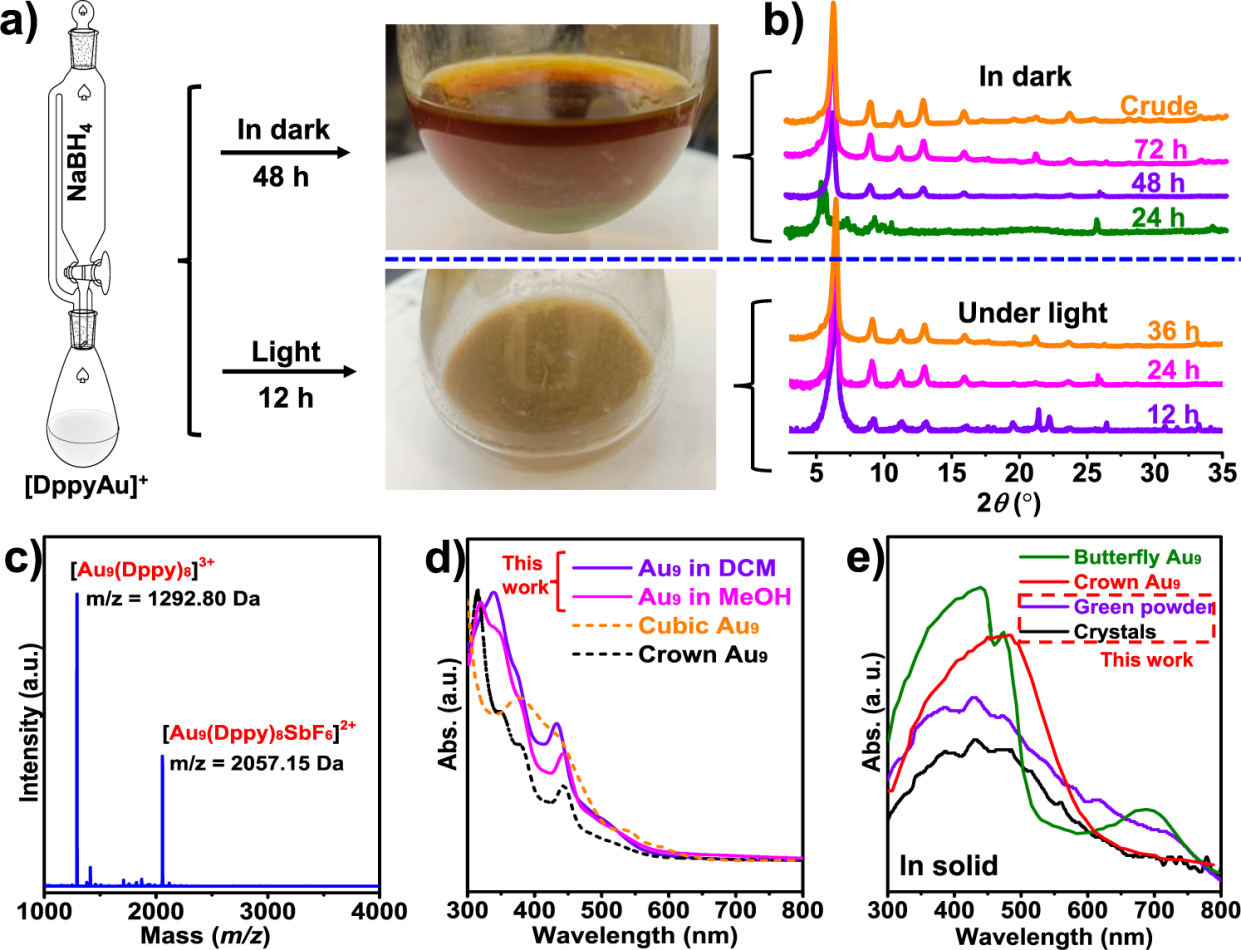
Synthesis and characterization of [Au_9_(Dppy)_8_]^3+^ cluster. (a) Synthetic route of the Au_9_ cluster. (b) Time-dependent XRD spectra during the formation of the Au_9_ cluster. (c) ESI-MS spectrum of the Au_9_ cluster. UV–vis spectra of the Au_9_ cluster: (d) in solution and (e) in solid.

Figure 1a presents the synthesis routine of these cubic Au_9_ clusters affected by light, which will influence the reduction process through a light-reduction process. A noteworthy observation is that light significantly influences cluster formation. It took 72 h to produce a green precipitate in darkness. However, upon light exposure, the reaction time could be shortened to 12 h to yield the same material. It is reasonable to assume that the light may accelerate the decomposition or transformation of metastable intermediate species, leading to the formation of stable Au_9_ clusters. This agrees well with the process described in the “size-focused” strategy proposed by Jin et al.^54^

To validate our hypothesis, we employed time-dependent X-ray diffraction technology to track the formation of the Au_9_ cluster. Before testing, we precipitated the solutes from the reaction solution by rapidly introducing them into hexane, thereby preserving their configurations and maintaining their structures as they existed in the solution.^55^ It was observed that the green powders formed in the reactor. These crude Au_9_ clusters were isolated from the reaction solution via centrifugation. As shown in Figure 1b, the Au_9_ cluster exhibits distinct XRD powder patterns at 6.5, 9.1, 11.2, 12.9, 15.9, and 23.6°. After 24 h, no precipitates were observed. The XRD spectrum of solutes has six pronounced peaks at 5.6, 5.8, 7.5, 9.5, and 10.7°, which differs from those of the target Au_9_ clusters, suggesting that no Au_9_ cluster forms yet. Green precipitate began to form in the reactor after a 48 h reaction, and the fresh precipitate generated from the top solution exhibited the same powder pattern as the Au_9_ cluster, indicating that the dominant species in the reaction solution became an Au_9_ cluster. After a 72 h reaction, plenty of green powder precipitated out. The solution turned light yellow, indicating the reaction termination point.

For comparison, it only needs 12 h to form the same green precipitate in the reactor under light (Figure 1b). The characteristic peaks of the Au_9_ cluster were detected in the powder pattern of solutes from the reaction solution after 12 h under light. With time going on, remarkable height enhancement of XRD peaks at 9.2 and 13.0° could be observed compared to the peak at 11.2°. The disappearance of unassigned peaks at 19.6, 21.4, 22.2, and 26.5° implies that some byproducts may transfer to the stable Au_9_ cluster. After approximately 36 h of reaction, the reaction solution turned to light yellow, signifying the progression of the reaction. The solutes in the reaction solution displayed the same powder pattern as the green precipitate, representing the completion of the reaction. All these results strongly corroborate our hypothesis that light plays a crucial role in promoting the transformation of metastable intermediates into the stable Au_9_ cluster.

Next, electrospray ionization mass spectroscopy (ESI-MS) was applied to identify the chemical composition and purity of the Au_9_ cluster. The obtained crude green powder was dissolved into dichloromethane and diluted with methanol to an appropriate concentration for the ESI-MS test. In the negative mode, no reasonable peaks were detected, whereas in the positive mode, several distinct peaks were observed, confirming that the gold nanoclusters were positively charged. As shown in Figure 1c, two pronounced peaks were detected at 1292.80 and 2057.15 Da, respectively. The most substantial peak at 1292.80 Da has a 0.33 Da (1/3 Da) space between its isotopic spectra (Figure S1a), suggesting that the peaks should be assigned to a +3 charged cluster ion. Thus, it can be determined to be [Au_9_(Dppy)_8_]^3+^ (1292.96 Da in theory), as their isotopic peaks align closely with the simulated values. Similarly, the latter peak at 2057.15 Da belongs to [Au_9_(Dppy)_8_·(SbF_6_)]^2+^ (2057.32 Da in theory, Figure S1b).

The UV–vis spectra of the Au_9_ cluster in solution and solid state were recorded and compared with previously reported Au_9_ clusters in other literature.^55–57^ Figure 1d presents the UV–vis spectra of the green powder, showing two sharp peaks at 339 and 433 nm, a shoulder peak at 373 nm, and a tail peak near 509 nm in CH_2_Cl_2_ solution (the deep-purple curve), which is different from that of the reported crown Au_9_ cluster (the black curve and Figure S2) and cubic Au_9_ cluster (the orange curve) in CH_2_Cl_2_.^56,57^ In the methanol solution, two sharp peaks at 319 and 443 nm and two shoulder peaks at 348 and 380 nm appear in the UV–vis spectra of the obtained Au_9_ cluster (the light-purple curve in Figure 1d), which is similar to that of the reported crown Au_9_ clusters.^46^ However, the curves of these two Au_9_ clusters differ in the peak positions and relative intensity. The results indicate that the motif of the newly synthesized Au_9_ cluster is different from those previously reported.

The solid UV–vis spectra further confirmed that the obtained Au_9_ clusters (single crystal and green powder) adopt a different motif from the reported crown-like and butterfly like Au_9_ clusters. As shown in Figure 1e, the purple and black curves represent the UV–vis spectra of the obtained Au_9_ cluster in powder and single crystals where its motif was determined (*vide infra*). Both curves display absorption peaks near 386, 430, and 475 nm on the scale of 300–600 nm range. It differs from the peak profiles of reported Au_9_ clusters,^44^ suggesting that the obtained Au_9_ cluster has a different structure from those reported in the solid state. Moreover, these characteristic peaks observed in both crystal and powder samples in this work indicate that the obtained Au_9_ clusters share the same motif in single crystals and green powder, as discussed later.

The results from ESI-MS and UV–vis spectra motivate us to reveal the crystal structure of the newly synthesized Au_9_ clusters in this study. Therefore, we analyzed the green single crystals generated from ether diffusion into its MeOH/CH_2_Cl_2_ solution through the single-crystal X-ray diffraction (SCXRD) technology, an important tool to identify the structures of materials at a molecular level. All the single crystals share the same shape (needle-like) and color (dark green). All these crystals are in a *Pn/3n* space group and share the same cell parameters (Table S1). These phenomena demonstrate that these single crystals have the same configuration and chemical compositions. The formula of the dark green crystals was determined as [Au_9_(Dppy)_8_]­(SbF_6_)_3_ by SCXRD, which is consistent with the result from ESI-MS. As depicted in Figure 2a, each cluster consists of nine Au atoms, forming an Au-centered cubic kernel, where a central Au atom is surrounded by eight lateral Au atoms located at eight vertices of a cubic. The metal core size is around 0.898 nm, corresponding to the sum of a radial Au–Au bond (2.703(3)) and a diameter of a Au atom (3.58 Å). This closely matches the values of the crown Au_9_ isomer (0.890 Å) and the butterfly Au_9_ isomer (0.900 nm).^58,59^ Consequently, the cubic Au_9_ cluster has a tighter and smaller core than the other Au_9_ clusters with crow and butterfly motifs. On the surface, eight Au–P bonds (2.301(5) Å) build up the connection between the gold core and phosphine ligands, forming the whole structure of the cubic Au_9_ cluster (Figure 2b,c). Notably, the surface Dppy ligands occupy the external regions of the cluster, creating a very tight protection layer for the metal kernel without any surface voids observed in other Au_9_ isomers.^58,59^ In summary, this compact arrangement and tight protective ligand layer make it structurally stable, an essential feature contributing to its overall stability compared to other isomers.

**Figure 2:**
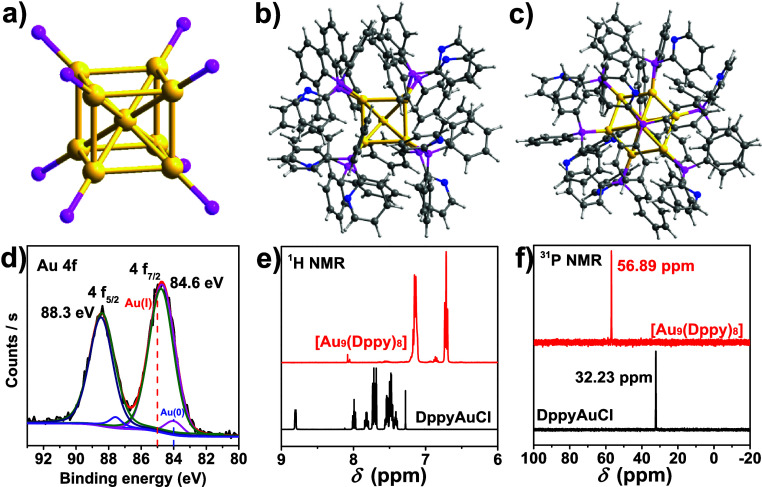
Physical textures of the cubic Au_9_ cluster. (a) Body-centered Au_9_ kernel and surface coordination model between gold kernel and phosphine ligands: (b) top view and (c) side view along the *C3* axis of symmetry. (d) Au 4f XPS scan. (e) ^1^H NMR and (f) ^31^P NMR spectra of the Au_9_ cluster and DppyAuCl in a CD_2_Cl_2_ solution. Color code: Au, yellow; P, purple, N, Blue; C, gray; H, white.

To identify the structure of the Au_9_ cluster in green powders, we compared the powder patterns generated from single crystals and green powders. As shown in Figure S3, the Au_9_ cluster in green powder shows a strong peak around 6.43°, which is the same with the simulated powder pattern from the crystal structure by SCXRD (6.44°). The other peaks also match those predicted ones, suggesting that the Au_9_ clusters in single crystals have the identical cubic motif. Moreover, the green powder obtained after the reaction without any purification showed the same powder patterns. Additionally, the powder pattern of the fresh Au_9_ cluster precipitated from the reaction solution also matches with those of the single Au_9_ cluster crystals. It illustrates that all these Au clusters have the same cubic structures. Therefore, this cubic Au_9_ cluster should have the same cubic motif in solution and in solid state (powder crystals or single crystals). These results agree with those from solid UV–vis spectra. Next, we investigated the compositions of Au_9_ clusters using X-ray photoelectron spectroscopy (XPS). Figure S4 shows that all the dominant elements, including Au, P, C, N, F, and Sb, were detected. The binding energies of Au 4f are ∼84.6 eV (Au 4f_7/2_) and 88.3 eV (Au 4f_5/2_) with a spin energy of 3.7 eV (Figure 2d). The binding energy of Au 4f_7/2_ is between the values of metallic Au^0^ (84.0 eV) and Au^I^ species (85.0 eV),^60,61^ suggesting that the Au species in the Au_9_ cluster is partially positively charged (Au^δ+^, 0 < δ < 1).^62^ This positive charge distribution is likely due to the coexistence of Au^0^ and Au^I^ within the metal core, where Au^0^ and Au^I^ correspond to the central Au atom and lateral Au atoms, respectively. To understand this statistical result in the Au_9_ kernel, one could imagine that nine Au atoms in the metal core share six free value electrons, causing a +3 charged Au_9_ cluster.

As described in its crystal structure, the Au–P bonds act as the bridge for the charge transfer between the metal kernel and surface Dppy ligands. Consequently, the metal core in the Au_9_ cluster influences the shielding effects of the H and P electrons. To explore this effect, we tested the ^1^H and ^31^P nuclear magnetic resonance (NMR) spectra of the Au_9_ cluster in CD_2_Cl_2_ solution (Figure 2e,f), which is compared with those of the DppyAuCl complex (Figures S5 and S6). The ^1^H NMR of DppyAuCl displays 7.40–8.80 ppm peaks, corresponding to the different protons (Figure S5) of phenyl and pyridyl rings, aligning well with theoretical values. The resonance peak of ^31^P is shown at 32.23 ppm as a singlet peak (Figure S6), which agrees with the reported work.^63^ Owing to the metal–ligand charge transfer (MLCT), the situation becomes complex for the Au_9_ cluster. As shown in Figure 2e and Figure S7, the dominant resonance peaks of protons shift to the high field. It suggests that the protons in Au_9_ experience a stronger electron shielding field compared to those in the DppyAuCl complex due to the metal-to-ligand charge transformation (MLCT). Moreover, the difference between protons of surface ligands also becomes exceedingly tiny, and only two prominent peaks at 6.71 and 7.15 ppm were detected, reflecting the high molecular symmetry of the Au_9_ cluster as observed by SCXRD. The high symmetry of the Au_9_ cluster is also reflected in its ^31^P NMR spectra since Figure 2f and Figure S8 only show a singlet peak at 56.89 ppm. This peak corresponding to the P atoms in Au_9_ ligands is closed to but different from that in [Au_9_(PPh_3_)_8_]­(PF_6_)_3_ (54.8 ppm) and [Au_9_(P­(p-MeC_6_H_4_)_3_)_8_]­(PF_6_)_3_ (53.5 ppm) previously reported.^63^ More importantly, the solo peak in ^31^P NMR spectroscopy indicates that the cubic motif is the only one in the CD_2_Cl_2_ solution, and it is very stable in CD_2_Cl_2_ since no decomposition products like DppyAuCl were detected.

### Stability and Reactivity of the Cubic Au_9_ Cluster

In the solid state, most nanoclusters protected by phosphine are typically stable, while in solution, their structures often undergo a unique reconstruction process, causing decomposition or aggregation. For example, the monovalent, metastable, and cubic Au_9_ cluster would transfer into Au_11_ within 90 min in CH_2_Cl_2_.^57^ The other Au_9_ clusters also show very high reactivity for their low stability in CH_2_Cl_2_.^53,55,64–66^ Therefore, the Au_9_ clusters were dissolved into CH_2_Cl_2_, which was then monitored by UV–vis spectroscopy under ambient conditions. Characteristic peaks of the Au_9_ cluster in CH_2_Cl_2_ solution remained unchanged for 8 h (Figure 3a); no noticeable changes were observed in the intensity of the peaks at 443 and 509 nm, which was related to the core structure of Au_9_ clusters. All these results suggest that this trivalent cubic Au_9_ cluster has high stability in the CH_2_Cl_2_ solution. It explains that it crystallizes from the CH_2_Cl_2_ solution for a relatively long time. Similarly, the stability of Au_9_ clusters was evaluated in MeOH solution under ambient conditions. As shown in Figure 3b, although the curves differ from those in DCM, they tend to overlap in 8 h, indicating that the cubic Au_9_ clusters also exhibit extremely high stability in methanol. Even though strong simulated sunlight was applied, this cubic Au_9_ cluster remained stable in the CH_2_Cl_2_/MeOH solution (Figure S9). These results confirm the robust nature of this trivalent cubic Au_9_ cluster in solution for prolonged periods, which is crucial for potential applications in catalysis and other solution-based processes, where maintaining the structural integrity of the nanocluster is essential for consistent performance. This could also open scenarios where structure manipulation could be done in a safe environment.

**Figure 3:**
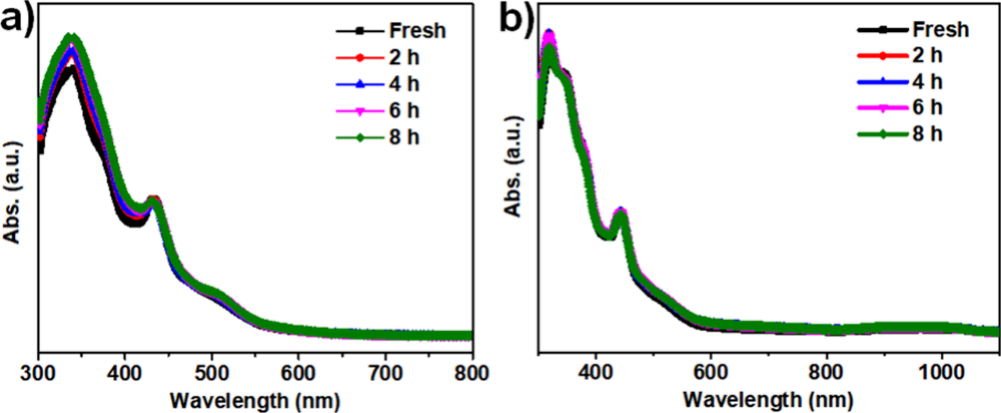
Stability of the cubic Au_9_ cluster in (a) DCM and (b) MeOH solutions. Of note, the increase of peaks in DCM was caused by the evaporation of solvents.

### Catalytic Performance of Gold Clusters in Lignin-Derived C–O and C–C Bond Cleavage

The subnanosized gold clusters, instead of bulk gold particles, demonstrate exceptional catalytic activity across various reactions, including oxidation, reduction, coupling reaction, biomass conversion, and CO_2_ fixation.^67–72^ However, gold nanoclusters have never been explored in the depolymerization of lignin due to the challenges in synthesizing gold nanoclusters and maintaining their subnanoscale size throughout the reactions.

Using the obtained cubic motif structure, we evaluated the catalytic performance of gold nanoclusters (cubic Au_9_ clusters) in the reductive depolymerization of lignin. To avoid clusters’ aggregation and reduce their loading levels, we diluted the Au_9_ cluster 100-fold with an acidic TiO_2_ supporter to form a precursor of Au_9_(Dppy)_8_/TiO_2_. This catalyst could turn into Au_9_/TiO_2_ by removing the organic ligands (according to the TGA of Au_9_ clusters, Figure S10) at 350 °C in N_2_ flow to expose more Au sites. Figure 4a illustrates that Au_9_/TiO_2_ exhibits similar XRD peaks with the TiO_2_ carriers. No obvious peaks assigned to Au_9_ clusters or gold particles were observed due to the very low loading ratio. Instead, the strongest peak was shifted to 25.37° (Au_9_/TiO_2_) from 25.42° (TiO_2_), and the other peaks remained unchanged (Figure S11). Furthermore, the full width at half-maximum (fwhm) widened to 0.29°, compared with TiO_2_ carriers (0.18°). The observed shift and broadened fwhm demonstrated the interaction between the small loading of Au clusters and the TiO_2_ supporter.^27^

**Figure 4:**
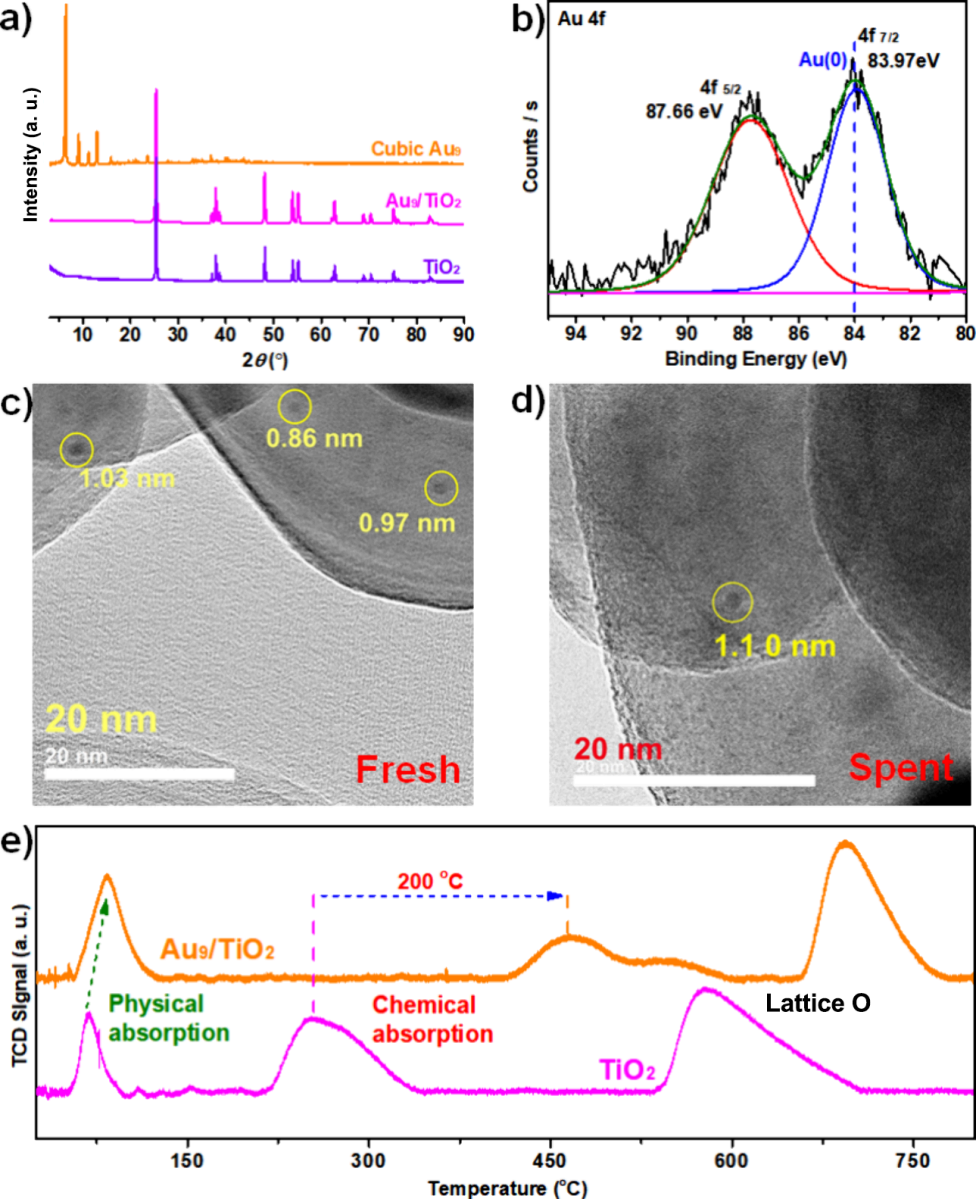
Characterization of Au_9_/TiO_2_ catalysts. (a) Powder patterns of TiO_2_, Au_9_ clusters, and Au_9_/TiO_2_. (b) High-resolution XPS analysis of Au_9_/TiO_2_. TEM images of (c) fresh and (d) spent Au_9_/TiO_2_ catalysts. The black spots are gold particles. (e) H_2_-TPD of Au_9_/TiO_2_ and TiO_2_.

Figure S12a shows dominant elements in the Au_9_/TiO_2_ catalyst, including Ti and O. No distinct peak corresponding to Au was observed due to its low loading ratio (<0.2% by XPS), while Au was detected in high-resolution scanning in Figure 4b. The binding energy of Au 4f orbitals was evaluated as 83.97 eV (4f_7/2_) and 87.66 eV (4f_5/2_) with a spin energy of 3.69 eV. A 0.63 eV negative shift was found in Au 4f_7/2_ compared to the free Au_9_(Dppy)_8_ (84.60 eV, Figure 2d), suggesting that the gold species in Au_9_/TiO_2_ should be reduced into metallic Au^0^ species on the surface of TiO_2_ carriers (84.0 eV in theory).^60,61^ Additionally, the reduction on Au species also reflected the strong metal–supporter interactions (SMSI)^73,74^ in the Au_9_/TiO_2_ catalyst. Moreover, the disappearance of P and N in high-resolution scans illustrated the removal of Dppy ligands from the Au_9_ cluster after annealing (Figure S12c,d). TEM images of Au_9_/TiO_2_ (Figure 4c) showed that the size of gold clusters is 0.86–1.03 nm, close to the size of free Au_9_(Dppy)_8_ clusters (∼0.89 nm). Thus, the anchored Au_9_ clusters should retain their core size during the removal of surface ligands. After the reaction, the size of the gold cluster is determined as ∼1.10 nm, slightly larger but still close to the core size of Au_9_ clusters, indicating that the gold clusters in Au_9_/TiO_2_ remain inert under reaction conditions.

We further investigated the H_2_ adsorption properties of TiO_2_ and Au_9_/TiO_2_ using temperature-programmed desorption (H_2_-TPD), where the desorption temperature reflects the adsorption energy of hydrogen and the nature of hydrogen bonding on the sample surface. As shown in Figure 4e, TiO_2_ exhibits three main desorption peaks at approximately 67, 255, and 578 °C. The first peak, at 67 °C, is attributed to physically adsorbed H_2_ due to its low adsorption energy. Chemically adsorbed H_2_, which binds more strongly, requires higher energy for desorption and thus appears around 255 °C. The third peak, beginning at 535 °C, is attributed to the loss of lattice oxygen from the TiO_2_ structure.

In comparison, the Au_9_/TiO_2_ catalyst shows a stronger H_2_ adsorption capacity, as evidenced by significantly temperature shifts in the TPD signal. The physical desorption of H_2_ under the Au_9_/TiO_2_ catalyst begins at 58 °C, about 6 °C higher than that under the TiO_2_ catalyst (52 °C). Hydrogen adsorption of the catalyst usually occurs between 200–400 °C. Herein, the chemical desorption of hydrogen of Au_9_/TiO_2_ starts at 418 °C, nearly double that of TiO_2_ (200 °C). This difference in chemical absorption reflected the influence of Au_9_ clusters on the property (like surface area, surface energy, and surface roughness, and surface vacancies) of the TiO_2_ supporter, as demonstrated by XRD. Additionally, a shoulder peak centered at 526 °C is likely due to hydrogen spillover and interaction with lattice oxygen from TiO_2_. The final desorption peak at 694 °C is assigned to the loss of lattice oxygen in the Au_9_/TiO_2_ catalyst, which is also much higher than that of TiO_2_. In summary, the hydrogen desorption of the catalyst Au_9_/TiO_2_ occurs at a higher temperature than that of TiO_2_, indicating that Au_9_/TiO_2_ exhibits stronger H_2_ adsorption than TiO_2_ alone, which is advantageous for high-temperature catalytic hydrocracking reactions.^75^

To investigate the catalytic performance of gold nanoclusters, we conducted experiments to depolymerize lignin dimer 1-(3,4-dimethoxyphenyl)-2-(2-methoxyphenoxy)­propane-1,3-diol (LD). This sample represents one of the lignin dimer models with three typical ubiquitous chemical bonds in natural lignin (C_α_–C_β_, C_β_–Cγ, and β*–*O–4 bonds).^2^ The dominant degraded products of LD were determined by GC-MS (Figures 5a). When the free Au_9_(Dppy)_8_ cluster was used, no products were detected, indicating that the free Au cluster was ineffective in degrading LD. Indeed, the subnanosized gold clusters could aggregate into bulky gold particles, causing inactivation. In contrast, the Au_9_(Dppy)_8_/TiO_2_ catalyst without annealing achieved a promising LD conversion of ∼92.42%, which is 6-fold higher than that of bare TiO_2_ (∼14.98%), shown in Table S2. The dominant products using the Au_9_(Dppy)_8_/TiO_2_ catalyst are determined as guaiacol (I, 92.03%) and 3,4-dimethoxytoluene (II, 59.66%) (Table S2). Additionally, other products like 3,4-dimethoxybenzaldehyde (VIII), 1,2-dimethoxy-4-vinylbenzene, 4-ethyl-1,2-dimethoxybenzene, 1-(3,4-dimethoxyphenyl)-3-hydroxypropan-2-one, (*E*)-1,2-dimethoxy-4-(prop-1-en-1-yl)­benzene, and 1-(3,4-dimethoxyphenyl)-3-hydroxypropan-1-one were detected with very low yields (Figure 5a), among which aldehydes and unsaturated products may act as intermediates (like 3,4-dimethoxybenzaldehyde) in the decomposition of LD. These findings suggest that the loaded Au_9_(Dppy)_8_ clusters play a crucial role in the depolymerization of LD, primarily facilitating the cleavage of the α-C–C and C–O bonds in the presence of Au_9_(Dppy)_8_/TiO_2_. However, a limitation of the Au_9_(Dppy)_8_/TiO_2_ catalyst is its single-use nature. The LD conversion rate dropped significantly to ∼18% in the second catalytic cycle. It likely stems from the decomposition or etching of Au_9_(Dppy)_8_ clusters from the TiO_2_ carrier under harsh reaction conditions, leading to the loss of active catalytic sites.

**Figure 5:**
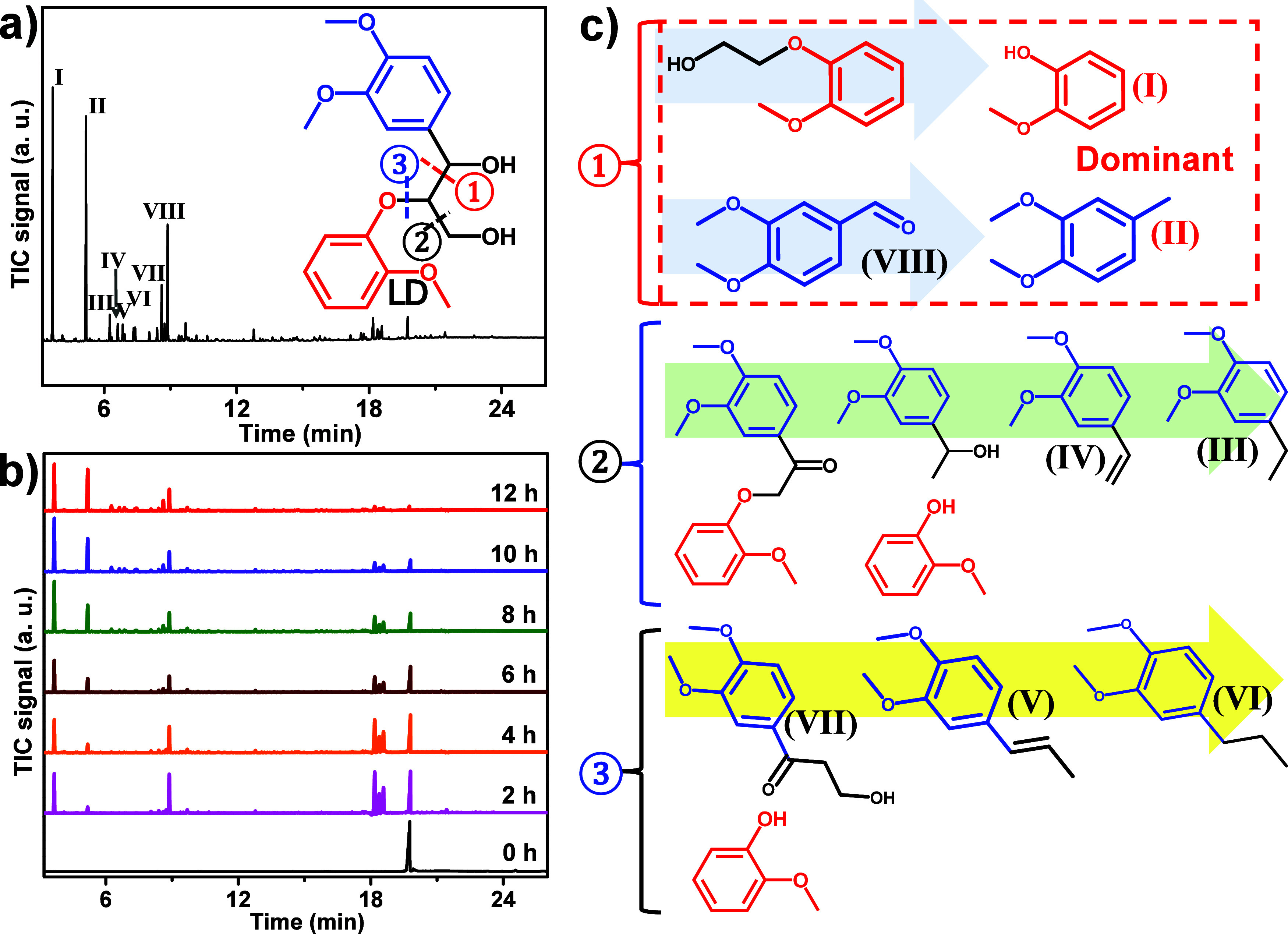
Decomposition of LD over Au_9_(Dppy)_8_/TiO_2_. Reaction conditions: 100 mg LD, 5 mg Au_9_(Dppy)_8_/TiO_2_ catalyst, 10 mL of THF, H_2_ (4 MPa), 400 °C, 12 h. The conversion of LD was determined by GC-FID using pure products as standards. (a) Products from LD decomposition over the Au_9_(Dppy)_8_/TiO_2_ catalyst. (b) Products from LD decomposition monitored by time-dependent GC-MS. (c) Three plausible reaction pathways of LD decomposition over the Au_9_(Dppy)_8_/TiO_2_ catalyst.

We have conducted the decomposition of LD over the Au_9_(Dppy)_8_/TiO_2_ catalyst along with the reaction time from 0 to 12 h. Figure 5b presents the product species change over time. During the decomposition of LD, the concentration of guaiacol (I) increases quickly, accompanied by the decrease of LD, indicating that LD would decompose into guaiacol rapidly over Au_9_(Dppy)_8_/TiO_2_ under the current reaction conditions. However, the concentration of 3,4-dimethoxytoluene (II) slowly increases as the reaction progresses, and its appearance is delayed compared to that of guaiacol. This suggests that 3,4-dimethoxytoluene is not directly transferred from LD, as that of guaiacol. Interestingly, the concentration of 3,4-dimethoxybenzaldehyde (VIII) in the reaction system increases rapidly for the first 2 h and then gradually decreases until the end of the reaction. Hence, 3,4-dimethoxybenzaldehyde may act as an intermediate during the decomposition of LD and finally transfers to other products. According to the final product composition and chemical structures, we propose that this intermediate transfers to 3,4-dimethoxytoluene, which exists in the final degraded products. Beyond 3,4-dimethoxybenzaldehyde, there are some other chemicals in the reactor exhibiting a similar tendency to 3,4-dimethoxybenzaldehyde, which should also be important intermediates during the decomposition of LD. The mass spectra of these species were determined as 316.13 Da, and they might be 1-(3,4-dimethoxyphenyl)-2-(2-methoxyphenoxy)­prop-2-en-1-ol, (*E*)-3-(3,4-dimethoxyphenyl)-2-(2-methoxyphenoxy)­prop-2-en-1-ol, and 1-(3,4-dimethoxyphenyl)-2-(2-methoxyphenoxy)­propan-1-one according to the current MS database in our system. Other byproducts like 1,2-dimethoxy-4-vinylbenzene, 4-ethyl-1,2-dimethoxybenzene, and 1-(3,4-dimethoxyphenyl)-3-hydroxypropan-2-one were also detected after an 8 h reaction and kept increasing until the end of the reaction. All these findings suggest that the decomposition of LD over Au_9_(Dppy)_8_/TiO_2_ under reaction conditions underwent different reaction pathways and produced the mixed degraded products at the end of the reaction.

To bring more insight into the decomposition mechanism of LD over the TiO_2_-carried nanogold catalysts, we deduced the formation of products from LD, as shown in Figure 5c. In pathway ①, LD could decompose into 2-methoxyphenyl (guaiacol), 3,4-dimethoxyphenylaldehyde, 3,4-dimethoxybenzyl alcohol, and 1,2-dimethoxy-4-methylbenzene through subsequent reduction reactions if the initial cleavage occurs at the α-C–C bonds. Pathway ② describes the scenario where the initial cleavage occurs at the γ-C–C bonds of LD, leading to the generation of 2-methoxyphenyl and aromatic products with two methoxy groups and a C2 carbon chain. If the aromatic products with a C3 carbon chain were observed, then it could be assigned to the products resulting from β*-*O-4 bond-cleavage-induced pathway ③. We noticed that the β*-*O-4 bond in LD will break finally to give guaiacol and that the 3,4-dimethoxytoluene (analogues) could be achieved by the cleavage of C_α_–C_β_ only. Hence, we could date back the accurate reaction happening to LD according to the final products over Au_9_(Dppy)_8_/TiO_2_. The C_α_–C_β_ bond-guided routine is the primary pathway (pathway ①) to give dominant products, guaiacol and 3,4-dimethoxytoluene (analogues). Two other pathways could happen to generate guaiacol and other byproducts but have very low probability. Therefore, Au_9_(Dppy)_8_/TiO_2_ will activate and break the C_α_–C_β_ bond in LD selectively under reaction conditions.

For comparison, we evaluated the catalytic performance of other homogeneous/heterogeneous gold clusters/particles. Figure 6a,b shows that the LD conversion into dominant products could achieve ∼67.20, 59.50, 47.3, 53.4, and 72.07% in the presence of [Au_9_(Ph_3_P)_8_]/TiO_2_, [Au_11_(Ph_3_P)_7_Cl_3_]/TiO_2_, [Au_13_(dppe)_10_Cl_2_]/TiO_2_ (dppe = 1,2-Bis­(diphenylphosphino)­ethane), [Au_25_(Ph_3_P)_10_Cl_8_]/TiO_2_, and [Au_13_Ag_12_(Ph_3_P)_10_Cl_8_]/TiO_2_ catalysts, respectively. Of note, all Au_9_, Au_11_, Au_13_, Au_25_, and Au_13_Ag_12_ clusters belong to the Au-centered nanoclusters with icosahedron-like metal cores (Figures S13 and S14).^76–79^ Crown-like [Au_9_(Ph_3_P)_8_] and [Au_11_(Ph_3_P)_7_Cl_3_] have open gold cores. [Au_13_(dppe)_10_Cl_2_] has a complete icosahedron gold core. [Au_25_(Ph_3_P)_10_Cl_8_] and [Au_13_Ag_12_(Ph_3_P)_10_Cl_8_] clusters share a similar metal core, a bi-icosahedron metal core by sharing an Au vertex, and surface ligands but different metal species. The lower LD conversion of [Au_9_(Ph_3_P)_8_] on TiO_2_ should result from the differences on ligand and gold core between [Au_9_(Ph_3_P)_8_] and [Au_9_(Dppy)_8_]. For example, the N in the [Au_9_(Dppy)_8_] cluster could form hydrogen bonds with hydroxyl groups in LD (N_pyridine_—H–O_LD_), enhancing the adsorption of LD on Au_9_ clusters beyond the π—π interaction between LD and surface ligands. Both [Au_9_(Ph_3_P)_8_] and [Au_11_(Ph_3_P)_7_Cl_3_] clusters are regarded as incomplete icosahedron clusters, and their core structures will affect the arrangement of surface ligands, which would directly influence the contraction of H_2_ and Au atoms in clusters.^71^ The tighter gold core in the [Au_11_(Ph_3_P)_7_Cl_3_] cluster gives it a closer ligand shell, leading to a further decrease in LD conversion rate because of the difficulty of H_2_ adsorption on Au atoms in the core; this inference has been confirmed by the further decrease in LD conversion rate when using [Au_13_(dppe)_10_Cl_2_]/TiO_2_ as a catalyst. A bigger [Au_25_(Ph_3_P)_10_Cl_8_] cluster, which is regarded as a fusion of two icosahedrons by sharing an Au vertex, might benefit for the decomposition of LD under hydro-processing, because the LD conversion has improved slightly. The recovery on LD conversion becomes obvious using [Au_13_Ag_12_(Ph_3_P)_10_Cl_8_] alloy clusters as catalysts, which should be attributed to the introduced Ag atoms in gold clusters, as observed in a previous report.^80^ However, the LD conversion went down to approximately 37.73% when the much bigger-sized Au NPs/TiO_2_ catalyst instead of the Au nanocluster was used, where the gold particle’s size was 3–5 nm (Figure S15), indicating that large-size gold may not be a good candidate for lignin depolymerization. Based on these results, we found that the catalyst efficiency of the TiO_2_-supported nanogold catalyst on the lignin conversion depends on the synergic effect of surface ligands as well, size distribution, topological structure, and chemical state of gold species. For instance, the LD conversion reached ∼78.52% using the Au_9_/TiO_2_ catalyst, where the gold clusters’ size was ∼1 nm. This conversion rate is lower than that using the Au_9_(Dppy)_8_/TiO_2_ catalyst but still over twice that using the Au NP/TiO_2_ catalyst. This result proves the synergic impact of surface ligands and particle size.

**Figure 6:**
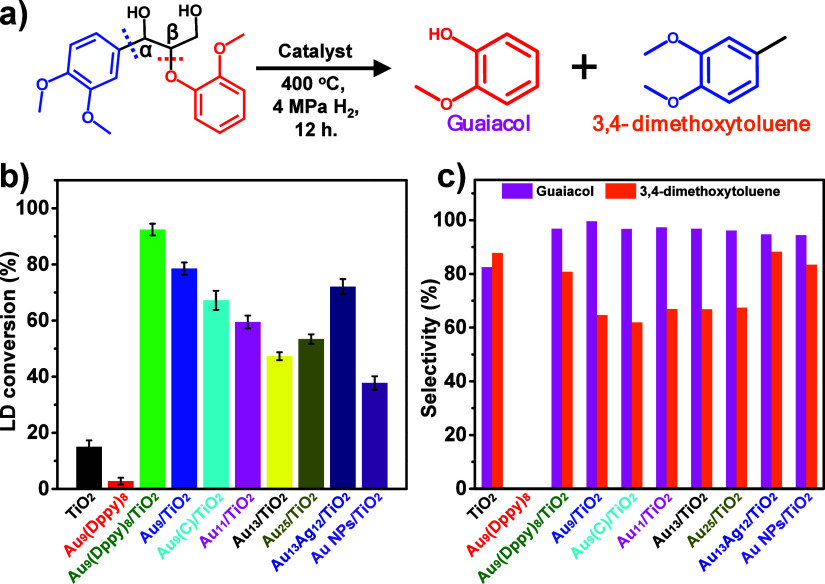
Catalytic performance of nanocluster-based catalysts in the depolymerization of the lignin model compound. (a) Conversion of the decomposition of LD into guaiacol and 3,4-bimethoxytolunene by cleavage of the C–C and C–O bonds. (b) Catalytic performances of nanogold-loaded catalysts in the decomposition of LD. (c) Selectivity of products from LD decomposition over different nanogold-loaded catalysts. ([Au_9_(Dppy)_8_]^3+^/TiO_2_, [Au_9_(Dppy)_8_]^3+^ on TiO_2_; Au_9_/TiO_2_, bare Au_9_ cluster on TiO_2_; Au_9_(C)/TiO_2_, crown-like [Au_9_(Ph_3_P)_8_]^3+^ on TiO_2_; Au_11_/TiO_2_, Au_11_(Ph_3_P)_7_Cl_3_ on TiO_2_; Au_13_/TiO_2_, Au_13_(Ph_3_P)_10_Cl_2_ on TiO_2_; Au_25_/TiO_2_, [Au_25_(Ph_3_P)_10_Cl_8_]^+^ on TiO_2_; Au_13_Ag_12_/TiO_2_, [Au_13_Ag_12_(Ph_3_P)_10_Cl_8_]^+^ on TiO_2_ (Reaction conditions: 100 mg LD, 5 mg Au_9_(Dppy)_8_/TiO_2_ catalyst, 10 mL THF, H_2_ (4 MPa), 400 °C, 12 h). The selectivity of guaiacol and 3,4-dimethoxytoluene is independent of each other.).

Moreover, we also evaluated the catalytic activity of Au_9_-based catalysts toward the cleavage of C–O and C–C bonds in LD by comparing the relative yields of guaiacol and 3,4-dimethoxytoluene (Figure 6c). Although the LD conversion differs according to catalysts, the yield of guaiacol is always higher than that of 3,4-dimethoxytoluene. This can be explained by three aspects. First, the C–O bonds are easier to cleave than the C–C bond, especially β-O-4 bonds, resulting in the production of abundant guaiacol. Second, another product, 3,4-dimethoxytoluene, needs to be formed through some intermediates, and more steps are required according to the previous reaction pathways shown in Figure 5. Third, other competitive byproducts can be formed through the pathway to produce 3,4-dimethoxytoluene, which further reduces its yield. Additionally, the relative yield of guaiacol and 3,4-dimethoxytoluene is closely related to the surface ligands of TiO_2_-supported Au_9_ nanoclusters. Though the dominant products and byproducts remain unchanged, the yield of 3,4-dimethoxytoluene rose to 80.8% from 64.55% (Au_9_(Dppy)_8_/TiO_2_) after removing surface ligands, catching up with the yield of guaiacol. Meanwhile, the yield of 3,4-dimethoxybenzaldehyde decreased as well. These findings suggest that the ligand-free Au_9_/TiO_2_ catalyst exhibits higher activity for the C–C bond cleavage than ligand-capped gold clusters by enhancing the reduction of 3,4-dimethoxybenzaldehyde into 3,4-dimethoxytoluene. This situation remains even if the reaction temperature decreased to 300 °C, where the LD conversion decreased to 43.73% (32.13% guaiacol, 24.61% for 3,4-dimethoxytoluene, and 5.47 for 3,4-dimethoxybenzaldehyde, Figure S16). Thus, it is safe to conclude that the surface ligands in Au_9_(Dppy)_8_/TiO_2_ significantly influence the pathway of LD decomposition. However, bare nanogold after the removal of ligands is likely beneficial for the cleavage of C–C bonds in lignin. Furthermore, we observed that the stability of the Au_9_/TiO_2_ catalyst was significantly enhanced as the maintained LD conversion rate remained around 76.27% after five reaction cycles (Figure S17). Hence, removing surface ligands from Au_9_ clusters should improve the interactions between Au_9_ and TiO_2_ supporters and avoid the loss of Au clusters from TiO_2_. Additionally, it is worth mentioning that the gold clusters in the Au_9_/TiO_2_ catalyst remained inert after the reaction, as shown in Figure 4d.

Finally, we further investigated the ligand effects on the catalytic activity of Au_9_-based catalysts by simulating the interaction between a ligand dimer model compound (LD) and Au_9_ clusters, both with and without surface ligands, using density functional theory (DFT) under ambient conditions. The DFT results suggest that the LD molecule can be readily physio-adsorbed onto the ligand-capped Au_9_ cluster, with an interaction energy of 0.95 eV (Figure S18). In contrast, the interaction energy decreases to 0.35 eV after the surface ligands are removed. This difference indicates that interactions between LD and the surface ligands—such as π···π, H···π, and hydrogen bonding (e.g., N_pyridine_···H­(O))—play a significant role in stabilizing the adsorption. Furthermore, the removal of surface ligands eliminates spatial hindrance between the Au_9_ core and the LD molecule, facilitating charge and [H] transfer among the support, cluster, and LD. This enhanced interaction likely explains the higher catalytic activity of bare Au_9_/TiO_2_ for C–C bond cleavage.

## Conclusions

In summary, we successfully synthesized the trivalent and body-centered gold nanocluster [Au_9_(Dppy)_8_]^3+^, filling the gap in crystal structures of the trivalent Au_9_ clusters. Using this Au_9_ cluster, we explored the potential applications of nanocluster-based catalysts in biomass conversion and lignin depolymerization in this work. This Au_9_ cluster is constructed by a cubic metal core capped with a tight monomolecular protection layer and exhibits the highest stability in CH_2_Cl_2_ compared with other Au_9_ clusters. The synthetic Au_9_ cluster was successfully deposited on nano TiO_2_ to give a nanocluster-based catalyst, where the nanoclusters maintain their subnanometer size. Compared with the other nanoclusters and nanoparticles, the TiO_2_-supported Au_9_ cluster with ligands shows the best catalytic performance (92.42% conversion) in the decomposition of the lignin model compound by breaking lignin-derived C–C and C–O bonds. Moreover, excellent durability could be achieved if the ligands were preremoved from clusters. The parallel experiments demonstrate that the size, shape, surface ligand, and metal chemical status play important roles in the catalytic activity. The ligand-capped nanoclusters show excellent activity to cleave the C–O bonds in lignin, and the bare gold nanocluster-based TiO_2_ catalyst, by removing the covered ligand, is beneficial for the cleavage of the C–C bonds in lignin. This study paves the way for designing high-performance metal nanocluster-based catalysts for lignin upgrading. Furthermore, for the first time, it introduces a novel strategy for tailoring clusters’ size, shape, surface ligand, and chemical environments of metal species to enhance the stability and catalytical efficiency of the nanocluster-based catalysts, with potential applicability to a broader range of reactions.

## Methods

### Synthesis of the Cubic [Au_9_(Dppy_3_)_8_]^3+^ Cluster

Generally, 100 mg of DppyAuCl (100 mg) was mixed with AgSbF_6_ (68.8 mg) in 8 mL of CH_2_Cl_2_/MeOH to give a colorless solution after filtration, which was then cooled to 0 °C for 30 min in an ice bath before the addition of NaBH_4_ (4 mg) dissolved into ice-cold EtOH (8 mL) dropwise. After 4-day stirring (or 2 days under light), Au_9_ clusters would be generated from the solution as a green precipitate. The green powder was isolated and washed with MeOH to remove byproducts. Au_9_ crystals were obtained as a deep-green crystal by the diffusion of ethyl ether into its CH_2_Cl_2_ solution for 2 weeks in the dark. Yield: 86.2 mg (∼84%, based on gold).

### Preparation of Metal Nanocluster-Loaded TiO_2_ Catalysts

TiO_2_ was heated in a vacuum oven at 150 °C for 4 h before use. The pretreated 1 g TiO_2_ was then dispersed into 30 mL of methanol forming a white suspension. Meanwhile, a 10 mg Au_9_ cluster was dissolved into 10 mL solvents of methanol dichloromethane (v/v = 1:1) to give a yellow-brown solution, which was then added into TiO_2_ suspension dropwise to generate a yellow mixture. After ∼2 h, the color of the mixture turned almost white, and the solid phase was collected by filtration. The Au_9_(Dppy)_8_/TiO_2_ catalyst was obtained as a light-gray powder after drying and activated in a vacuum oven at 150 °C.

Au_9_(Ph_3_P)_8_, Au_11_(Ph_3_P)_7_Cl_3_, Au_13_(dppe)_5_Cl_2_, Au_25_(Ph_3_P)_10_Cl_8_, and Au_13_Ag_12_(Ph_3_P)_10_Cl_8_ clusters were prepared according to the reported methods^76–79^ and loaded on TiO_2_ to give Au­(C)/TiO_2_, Au_11_/TiO_2_, Au_13_/TiO_3_, Au_25_/TiO_2_, and Au_13_Ag_12_/TiO_2_ catalysts. The Au_9_(Dppy)_8_/TiO_2_ catalyst was heated to 350 °C to remove surface ligands to prepare the Au_9_/TiO_2_ catalyst.

### Preparation of Gold Nanoparticle-Loaded TiO_2_ Catalysts

Typically, 5 mg of chloroauric acid was dissolved into 10 mL of water and mixed with 200 mg of TiO_2_. After 10 min of ultrasonication, 0.1 M NaOH solution was added into the above mixture dropwise (0.5 drop per second) until pH 8 and under vigorous stirring. After another 2 h, the obtained white mixture was collected and washed with deionized water to remove soluble salts. TiO_2_ powder was then heated to 300 °C under a 5% H_2_–N_2_ flow for 3 h. The obtained sample was labeled as Au NPs/TiO_2_.

### Synthesis of 1-(3,4-Dimethoxyphenyl)-2-(2-methoxyphenoxy)­propane-1,3-diol

A lignin dimer was synthesized according to the method reported in the literature.^14^ As illustrated in Scheme 1, guaiacol (6.2 g) and potassium carbonate (7.6 g) were mixed in 50 mL of acetonitrile, followed by addition of ethyl bromoacetate (9.124 g). After 24 h of reflux at 80 °C, P1 was formed in a yellow solution, which was purified by silica gel using petroleum as eluent. Under the protection of N_2_, the purified and dried P1 was dissolved into 50 mL of tetrahydrofuran (THF) and cooled to −78 °C, followed by the addition of lithium diisopropylamide (LDA) (7 mL, 2 M solution) at −78 °C dropwise to give a red solution. After 15 min, 3.0 g of veratrylaldehyde was dissolved in 20 mL of dried THF and added into the reactor at −78 °C dropwise. After a 2 h reaction, hydrochloric acid was used to quench the reaction to give a yellow solution. A gradient silica gel was applied to purify the product P2 using ethyl acetate and petroleum as eluent. The final product LD was obtained as yellow after reduction of NaBH_4_ and silica gel purification. ^1^H NMR (CDCl_3_: 7.28 ppm): 6.5–7.3 ppm (m, Ar-H, 7H), 4.65 ppm (d, C_α_-H, 1H), 4.24 ppm (m, C_β_-H, 1H), 4.14 (d, Cγ-H,1H), 4.06 (d, Cγ-H, 1H), 3.91 (s, O–CH_3_, 9H).

**Scheme 1:**
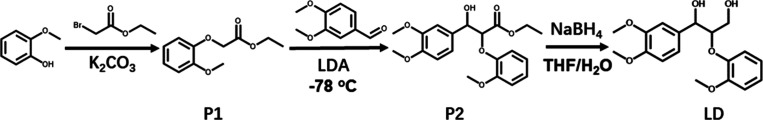
Routine for the Synthesis of LD

### General Method for the Decomposition of Lignin

In a run, 100 mg of lignin dimer was mixed with 20 mg of catalyst in 10 mL of THF and sealed in a 50 mL Parr reactor at 400 °C under 4 MPa H_2_ for 12 h. Upon completion, the reactor was cooled to room temperature before pressure release in a fume hood. The obtained mixture, including catalyst and reactor cleansing solution, was transferred and diluted to 15 mL with THF in a 50 mL centrifuge. After centrifugation (5000 rpm, 5 min), 2 mL of top clear solution was filtered into a sample vial for GC-MS (5 μL) and GC-FID (10 μL) test. The products of the lignin dimer were determined by the GC-MS system. Moreover, the concentration of each compound in the solution was calculated based on the corresponding calibration curve based on a series of standard solutions of the same compounds. The total conversion of lignin dimer and the yield of products were calculated based on GC-FID according to eqs 1 and 2.

$$Y\left(\mathrm{LD}\right)=\frac{C_{0}-C^{\prime }}{C_{0}}\times 100\%$$ 1

*Y*(LD) is the total conversion of the lignin dimer, Co is the original concentration of the lignin dimer (∼6.67 mg/mL), and *C*′ is the concentration of the lignin dimer after reaction (mg/mL).

$$Y\left(p\right)=\frac{15\mathrm{mL}\times C\left(p\right)÷M\left(p\right)}{15\mathrm{mL}\times C_{0}÷M\left(\mathrm{LD}\right)}\times 100\%$$ 2

*Y*(*p*) is the yield of the product, *C*(*p*) is the concentration of the product after reaction (mg/mL), *M*(*p*) is the molecular weight of the product, and *M*(LD) is the molecular weight of the lignin dimer (334.14 g/mol).

### Characterization

UV–vis was applied on an AOE 1900-PC spectrometer. Crystal samples were dissolved into dichloromethane and diluted to the proper concentration before being tested. Electrospray ionization mass spectrometry (ESI-MS) was conducted on an UltiMate 3000 RSLC Nano UHLPC-Q Exactive Plus Hybrid quadrupole-orbitrap mass spectrometer using methanol as the liquid phase. Samples were dissolved in CH_2_Cl_2_ and diluted into suitable concentrations before being tested. The X-ray diffraction (XRD) measurements were carried out on a Rigaku MiniFlex Powder X-ray Diffractometer with monochromatic Cu Kα radiation (λ = 1.5418 Å) at the voltage and current of 40 kV and 250 mA, respectively. 2θ was scanned over the range of 3–90° at a rate of 10 K/min. The surface composition and surface electronic state were analyzed by X-ray photoelectron spectroscopy (XPS) using a Thermo K-Alpha XPS instrument at 160 eV pass energy. Al Kα radiation was used to excite photoelectrons. The binding energy value of each element was corrected using C 1s = 284.8 eV as a reference. Hydrogen temperature-programmed desorption (H_2_-TPD) was conducted in a self-built tube furnace-GC system equipped with a thermal capability detector (TCD) using pure nitrogen as a carrier. ∼150 mg samples were used and pretreated in a nitrogen flow (50 mL/min) at 250 °C for 4 h to remove adsorbed small molecules like CO_2_ and H_2_O. A continuous 10% H_2_–N_2_ flow (50 mL/min) was charged for 1 h at 25 °C to research the H_2_ adsorption saturation state. After cleaning the whole system with pure nitrogen for 2 h, the temperature increased to 800 °C from 25 °C at a 5 K/min ramping rate and GC/TCD was performed to record the hydrogen spillover simultaneously in a nitrogen flow of 50 mL/min.

### X-ray Crystallography

A single-crystal sample was selected and collected on a Bruker APEX-II CCD diffractometer. The crystal was kept at 173.06 K during data collection. Data collection and reduction were performed on CrysAlis^*pro*^ packages. The structure was solved by SHELXT^81^ and refined by SHELXL^82^ on the package of Olex2. Refined details are listed in Table S1. The residual electron density peaks around the lateral Au atoms were treated as disordered. B-level warnings related to P1 atom and Sb1 atom are explained and caused by the disorder on the *C3* axel, which will not affect the structure model of the Au_9_ cluster.

Crystal data for C_136_H_112_Au_9_F_18_N_8_P_8_Sb_3_ (CCDC: 2429416): cubic, *Pn*3̅*n* (no. 222), *a* = 18.99320(10) Å, *V* = 6851.64(11) Å^3^, *Z* = 2 *T* = 173.06 K, 48075 reflections, final *R*_1_ = 0.1028 (*I* > 2σ­(I)), *wR*_2_ = 0.2380, *S* = 1.313.

### Gas Chromatography–Mass Spectrometry (GC-MS)

The products of the lignin dimer were determined by the mass spectra obtained from an Agilent 7820/5977 GC-MS system. Helium was used as the carrier gas at a flow rate of 1 mL/min with a 1:50 split ratio. An Agilent HP-5MS 5% Phenyl Methyl Silox 30 m × 250 μm × 0.25 μm column was set in the GC oven to separate the sample. Moreover, the oven temperature ramped up from 50 to 280 °C at a rate of 10 °C/min. The energy of the electrons was set at 70 eV to generate MS spectra comparable with the online database.

### Gas Chromatography-Flame Ionization Detector (GC-FID)

The yield of products of the lignin dimer was evaluated through an HP 6890 GC-FID system equipped with an Agilent HP-5MS 5% Phenyl Methyl Silox 30 m × 250 μm × 0.25 μm column in the GC oven. Nitrogen was used as a carrier gas at a flow rate of 1 mL/min with a 1:50 split ratio. Moreover, the oven temperature ramped up from 50 to 280 °C at a rate of 10 °C/min.

### DFT Methodology

Spin-polarized DFT calculations were performed using the Vienna *Ab initio* Simulation Package (VASP).^83,84^ All nanoclusters and nanocluster complexes were optimized in a cubic box surrounded by ∼15 Å of vacuum, with initial structures taken from crystallographic information files. For the exchange–correlation energy, the Perdew–Burke–Ernzerhof (PBE) version of the generalized gradient approximation (GGA) was used.^85^ The ion–electron interaction was described by a projector-augmented wave (PAW),^86^ and the wave function was expanded by plane waves with a cutoff energy of 400 eV. Geometry relaxations were performed using the conjugate gradient algorithm until the residual force components on each atom were <0.03 eV/A. The Γ point alone was used to sample the *k*-space.

## Supplementary Material



## Data Availability

Crystallographic data for the [Au_9_(Dppy_3_)_8_]^3+^ cluster (CCDC number: 2429416) can be obtained free of charge via www.ccdc.cam.ac.uk/data_request/cif, by emailing data_request@ccdc.cam.ac.uk, or by contacting The Cambridge Crystallographic Data Centre, 12 Union Road, Cambridge CB2 1EZ, UK; fax: + 44 1223 336033.

## Notes

Materials; isotopic peaks of the [Au_9_(Dppy_3_)_8_]^3+^ cluster (S1); UV–vis spectra of the [Au_9_(PPh_3_)_8_]^3+^cluster cluster (S2); X-ray diffraction spectra of the [Au_9_(Dppy_3_)_8_]^3+^ cluster (S3); X-ray photoelectron spectra (S4); nuclear magnetic resonance (S5–S8); stability of the [Au_9_(Dppy_3_)_8_]^3+^ cluster in methanol (S9); thermogravimetric analysis of the [Au_9_(Dppy_3_)_8_]^3+^ cluster (S10); X-ray diffraction spectra of the TiO_2_-supported [Au_9_(Dppy_3_)_8_]^3+^ cluster; X-ray photoelectron spectra of the TiO_2_-supported [Au_9_(Dppy_3_)_8_]^3+^ cluster without ligands (S12); products from LD (S13); plausible pathways for the LD decomposition (S14); TEM of Au NPs/TiO_2_ (S15); durability test of Au_9_/TiO_2_ (S16); crystal data and structure refinement for the cubic Au_9_(Dppy)_8_ cluster (Table S1); and catalyst performance (Table S2) (PDF)
