# Supplementary material for: A Robust Atomically Precise Nanocluster Catalyst for Simultaneous C–O and C–C Bond Cleavage in Lignin Models
Source: ACS Catal. 2025 Nov 24;15(23):20270–83. doi: 10.1021/acscatal.5c07479 (PMC12687381; doi:10.1021/acscatal.5c07479)
Supplement: Supplementary file 1 [file cs5c07479_si_001.pdf]

Supporting Information

A Robust Atomically Precise Nanocluster Catalyst for Simultaneously C-O and C-C bond Cleavage in Lignin Models

Zhaoxian Qin,[a,b] *Akanksha Lakra,*[a] *Rahul R Somni,*[c] *Wenbo Peng,*[a] *Gao Li*[b] *Guoxiang Hu,*[c] *Zhaohui Tong* [a, *]

[a] School of Chemical and Biomolecular Engineering, Georgia Institute of Technology, Atlanta, GA. 30332 USA. Email: zt7@gatech.edu (Z.T.)

[b] School of Chemistry and Chemical Engineering, Inner Mongolia Normal University, Hohhot 010018, China.

[c] School of Materials Science and Engineering, Georgia Institute of Technology, Atlanta, GA. 30332 USA.

[*]Corresponding author: Zhaohui Tong (zt7@gatech.edu)

Contents

Materials 2

Supporting Figures 3

Supporting Tables 13

Materials

DppyAuCl, $AgSbF_6$, $NaBH_4$, dichloromethane (DCM), methanol (MeOH), ethanol (EtOH), and ethyl ether were purchased from Thermo Fisher Scientific. All the chemicals and solvents were used as received without further purification. TiO_2 powder was reduced at 700 ^{o}C under hydrogen gas for 3 hours before use. Lignin dimer (LD) and Meatal nanoclusters, including $[Au_9(PPh_3)_8]^{3+}$, $Au_{11}(PPh_3)_7Cl_3$, and $Au_{13}Ag_{12}(PPh_3)_{10}Cl_8$ mentioned in this work were prepared in our lab according to the previously-reported methods.[1-3] All the glassware was washed with aqua regia and deionized water before use.

Supporting Figures

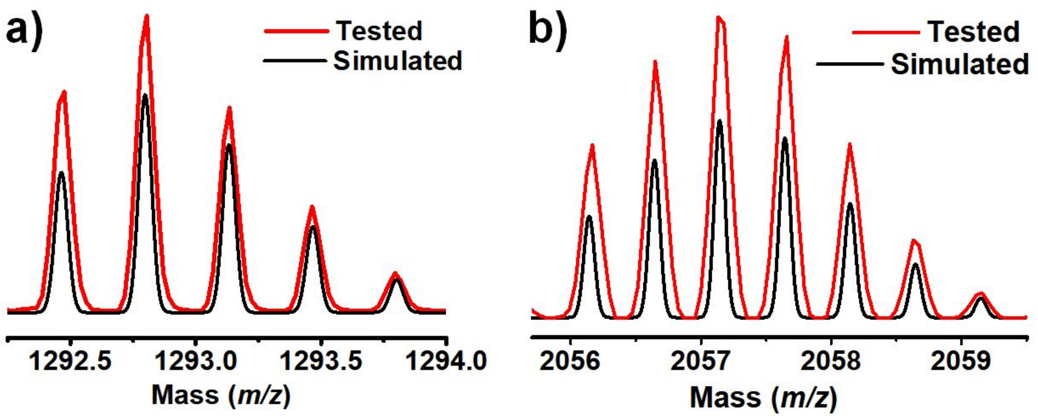

Figure S1. The isotopic peaks of $[Au_9(Dppy)_8SbF_6]^{2+}$ fragment ions compared with the simulated peaks. (a) +3 charged peak. (b) +2 charged peak.

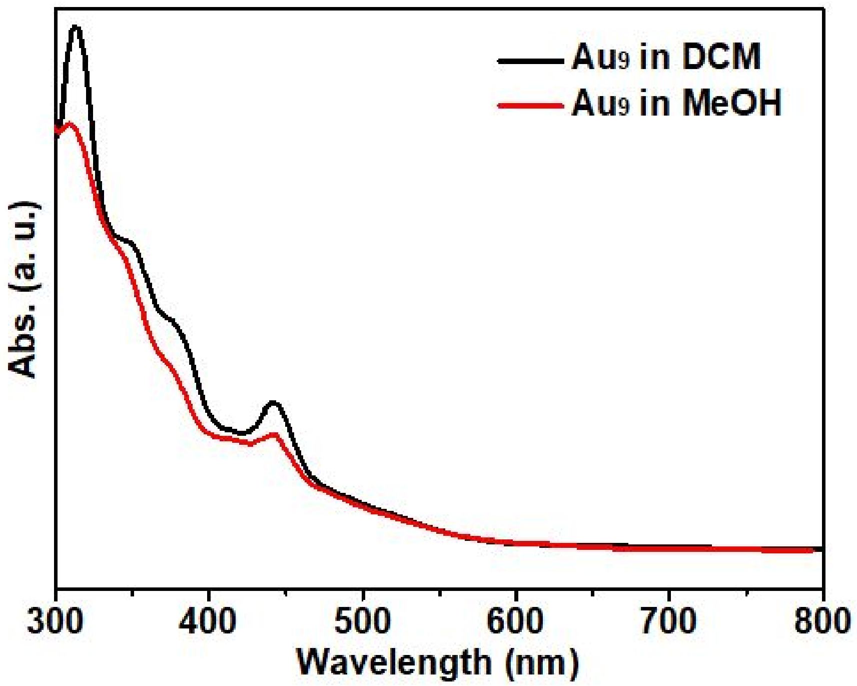

Figure S2. UV-vis spectra of reported $[Au_9(Ph_3P)_8](SbF_6)_3$ clusters in different solvents.

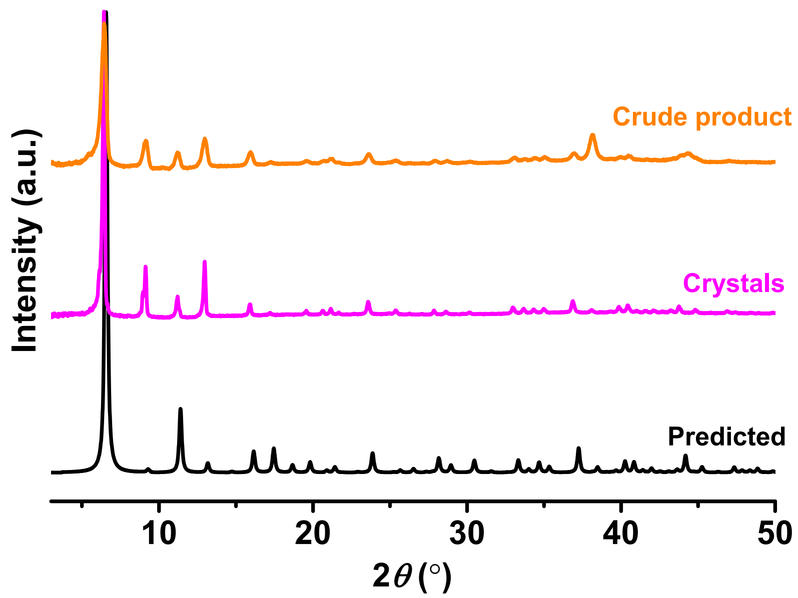

Figure S3. Powder patterns of Au_9 clusters in different statuses. (Black curve: predicted form single crystal structure; Pink curve: single crystal samples; Orange curve: crude product in powder)

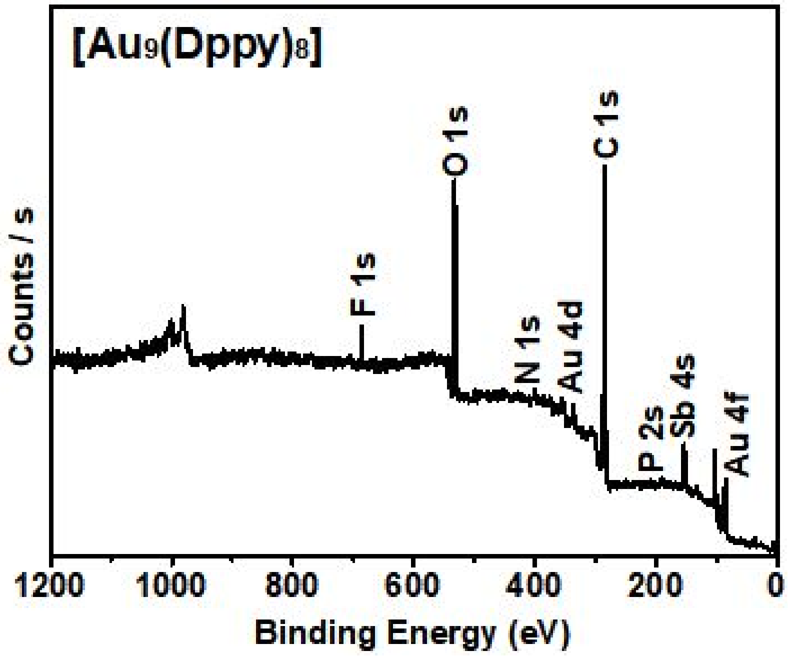

Figure S4. XPS survey scan of Au_9 cluster.

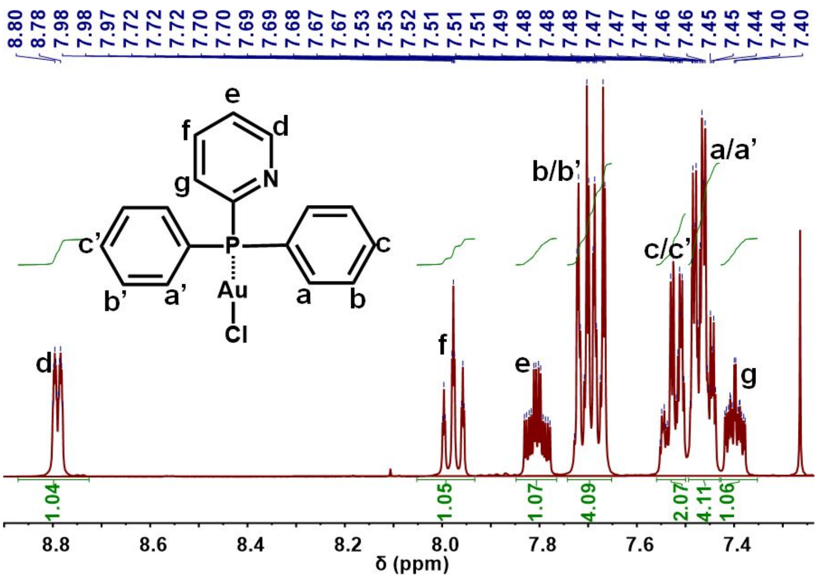

Figure S5. ^{1}H NMR of DppyAuCl clusters in $CDCl_3$.

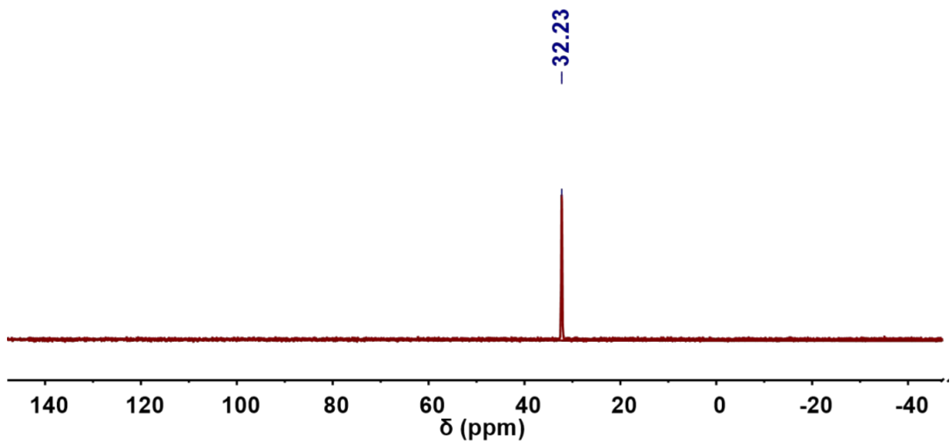

Figure S6. ^{31}P NMR of $[Au_9(Dppy)_8]$ clusters in $CDCl_3$.

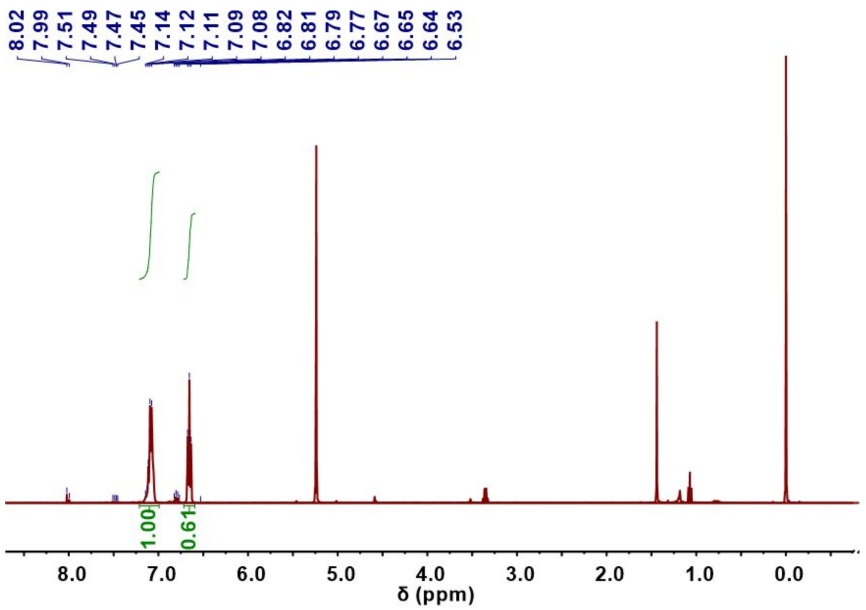

Figure S7. ^{1}H NMR of $[Au_9(Dppy)_8]$ clusters in CD_2Cl_2 (CD_2Cl_2: 5.28 ppm).

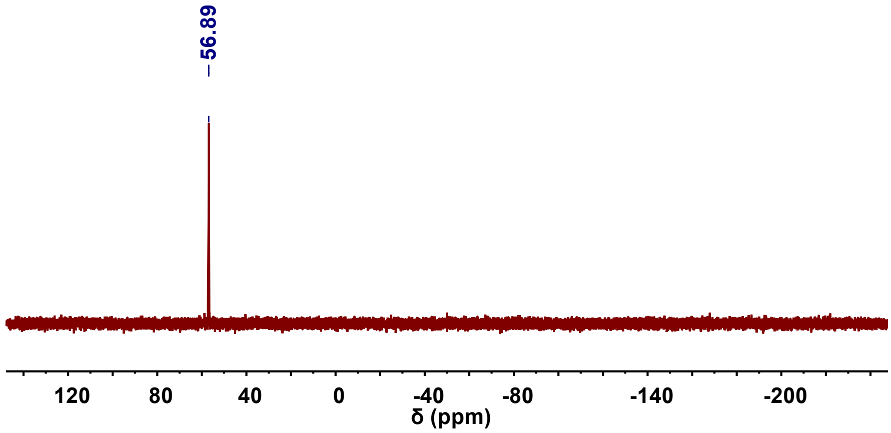

Figure S8. ^{31}P NMR of $[Au_9(Dppy)_8]$ clusters in CD_2Cl_2.

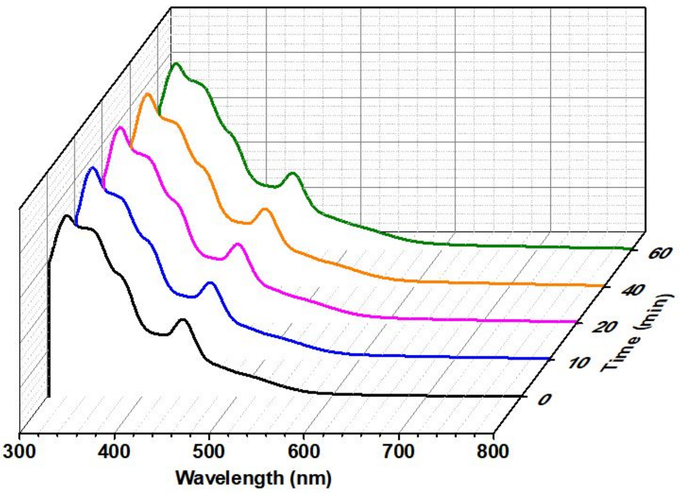

Figure S9. Stability test of the Au_9 cluster in DCM/MeOH solution under simulated sunlight.

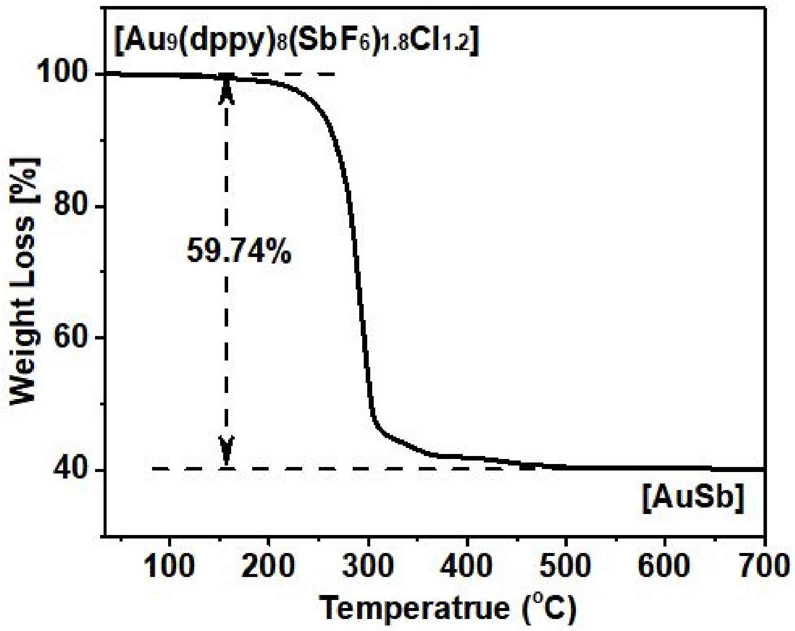

Figure S10. TGA result of Au_9 clusters under N_2 atmosphere. TGA result of Au_9 clusters shows that all the surface organic ligands (Dppy and Cl) are detached at ~ 350 °C.

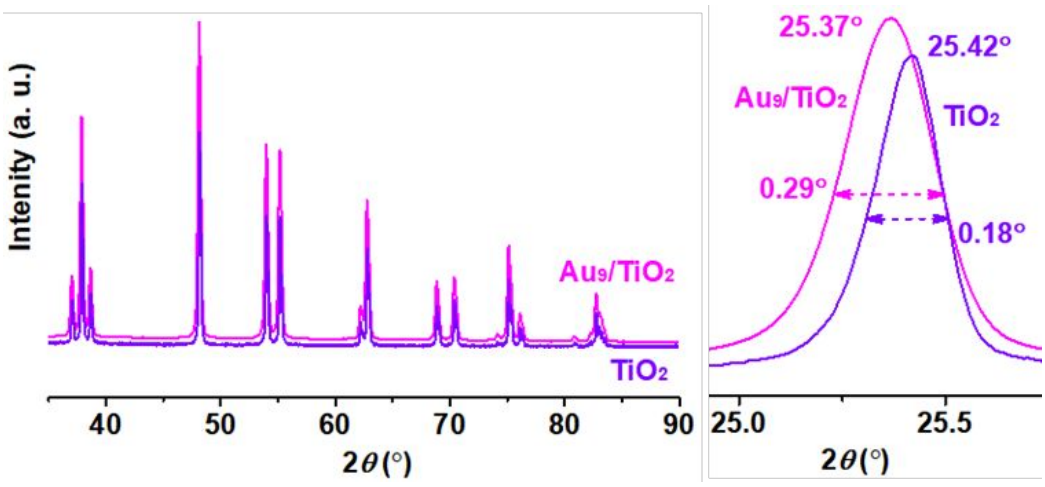

Figure S11. High-resolution XRD spectra of catalysts.

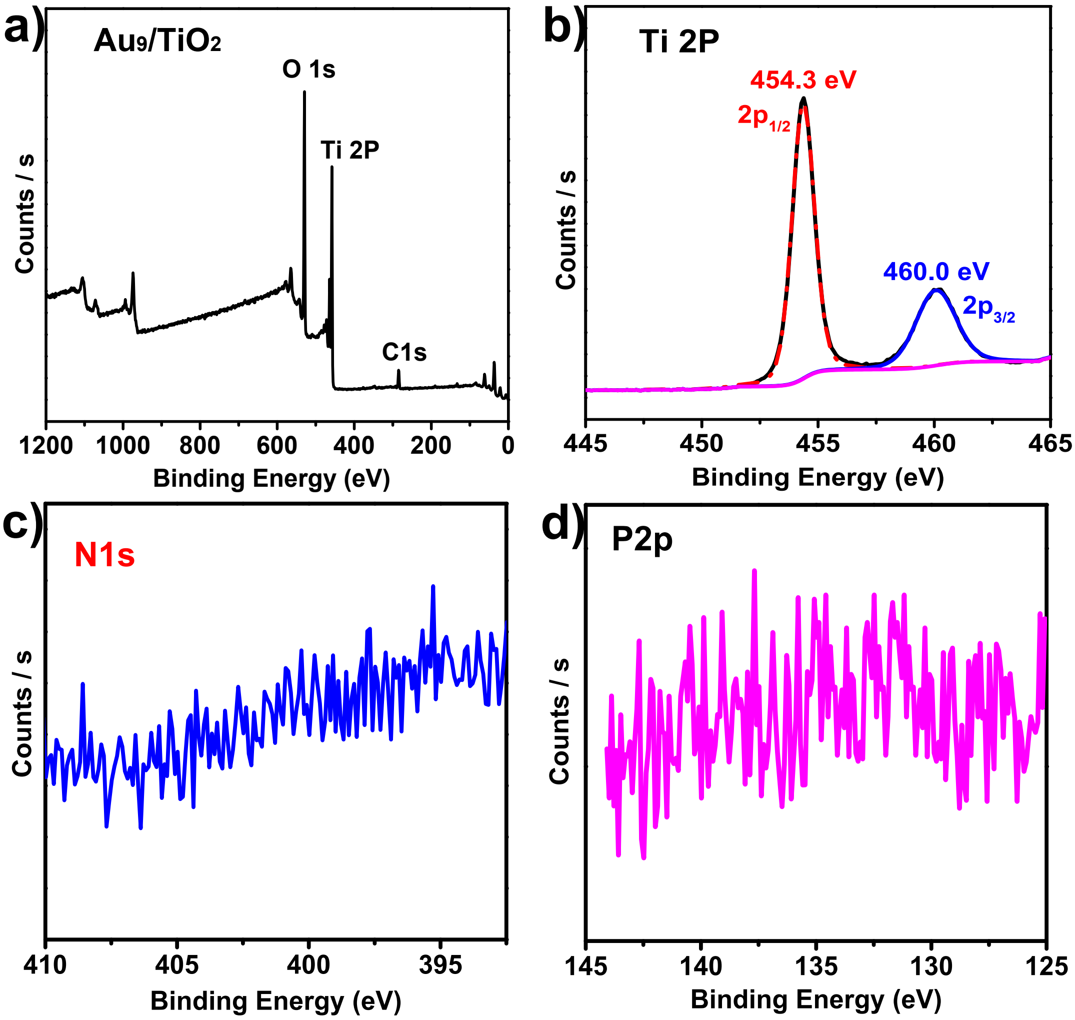

Figure S12. XPS spectra of Au_9/TiO_2: (a) Full survey scan, (b) Ti 2P, (c) N 1s, and P 2p.

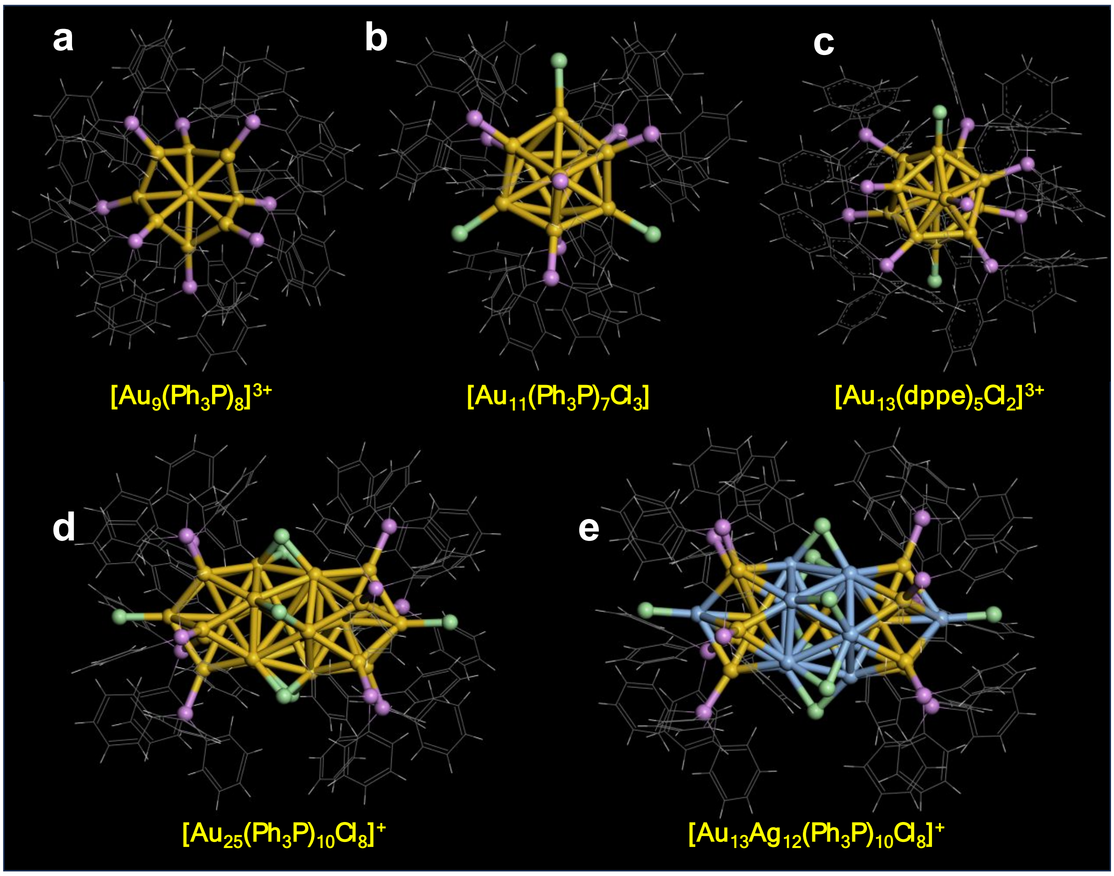

Figure S13. Crystal structures used in this work. (a) Crown-like $[Au_9(Ph_3P)_8]^{3+}$. (b) $Au_{11}(Ph_3P)_7Cl_3$. (c) $[Au_{13}(dppe)_{10}Cl_2]^{3+}$(dppe = 1,2-Bis(diphenylphosphino)ethane). (d) $[Au_{25}(Ph_3P)_{10}Cl_8]^{+}$. (e) $[Au_{13}Ag_{12}(Ph_3P)_{10}Cl_8]^{+}$.

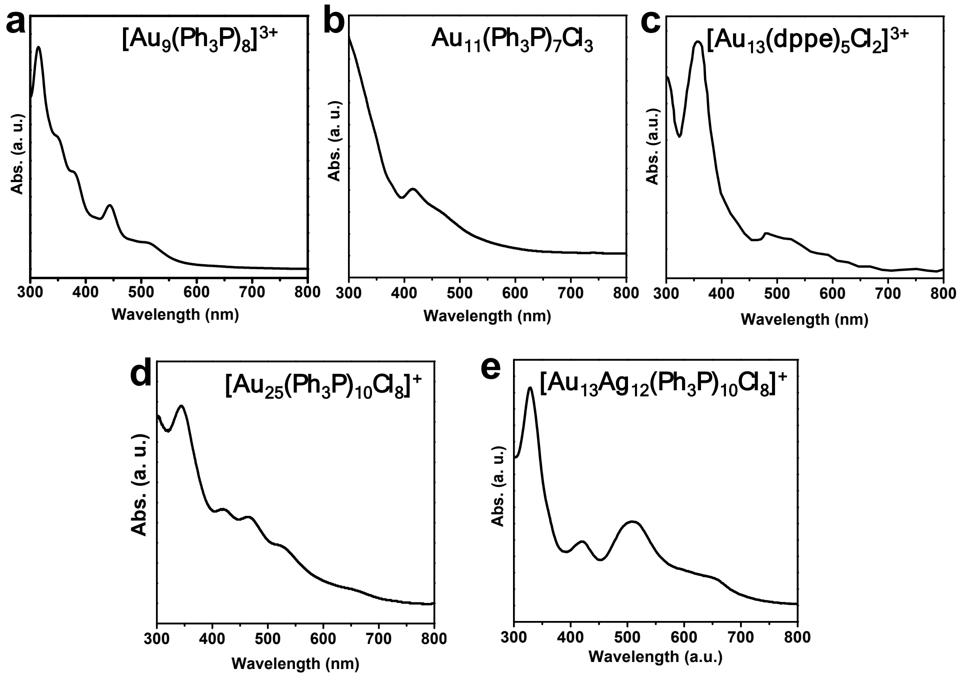

Figure S14. UV-vis spectroscopy of metal nanoclusters used in this work. (a) Crown-

like $[Au_9(Ph_3P)_8]^{3+}$. (b) $Au_{11}(Ph_3P)_7Cl_3$. (c) $[Au_{13}(dppe)_{10}Cl_2]^{3+}$(dppe = 1,2-Bis(diphenylphosphino)ethane). (d) $[Au_{25}(Ph_3P)_{10}Cl_8]^{+}$. (e) $[Au_{13}Ag_{12}(Ph_3P)_{10}Cl_8]^{+}$.

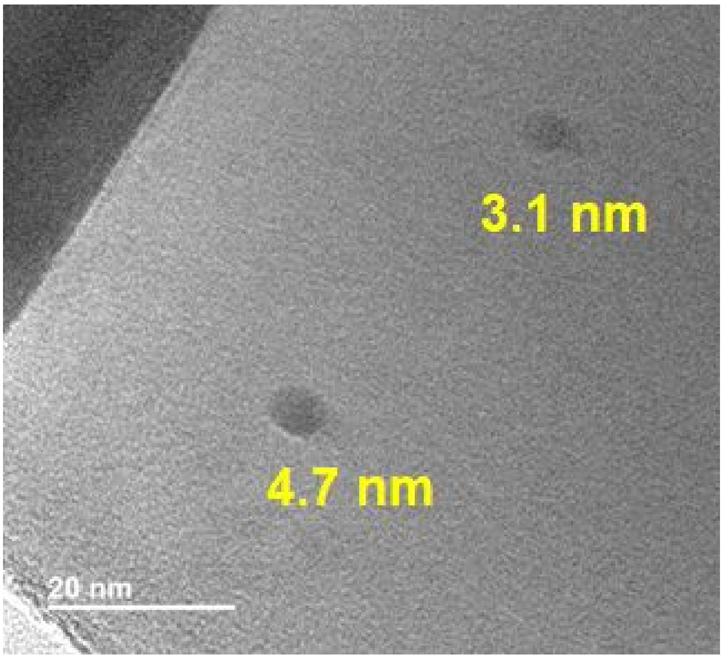

Figure S15. TEM image of Au NPs/TiO_2 catalyst.

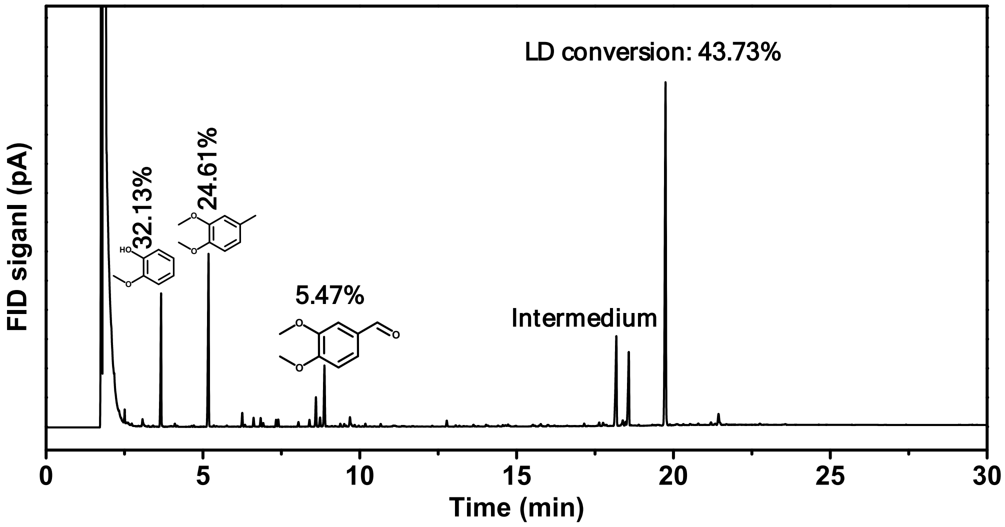

Figure S16. The yields of guaiacol and 3,4-dimethoxytoluene at a lower LD conversion, which represented the cleavage of C-O and C-C bonds in lignin dimer model compound, respectively. (Reaction conditions: 100 mg LD, 5 mg catalyst, 10 mL THF, H_2 (4 MPa), 300 °C, 12 h. The conversion of LD was determined by GC-FID using pure products as standards)

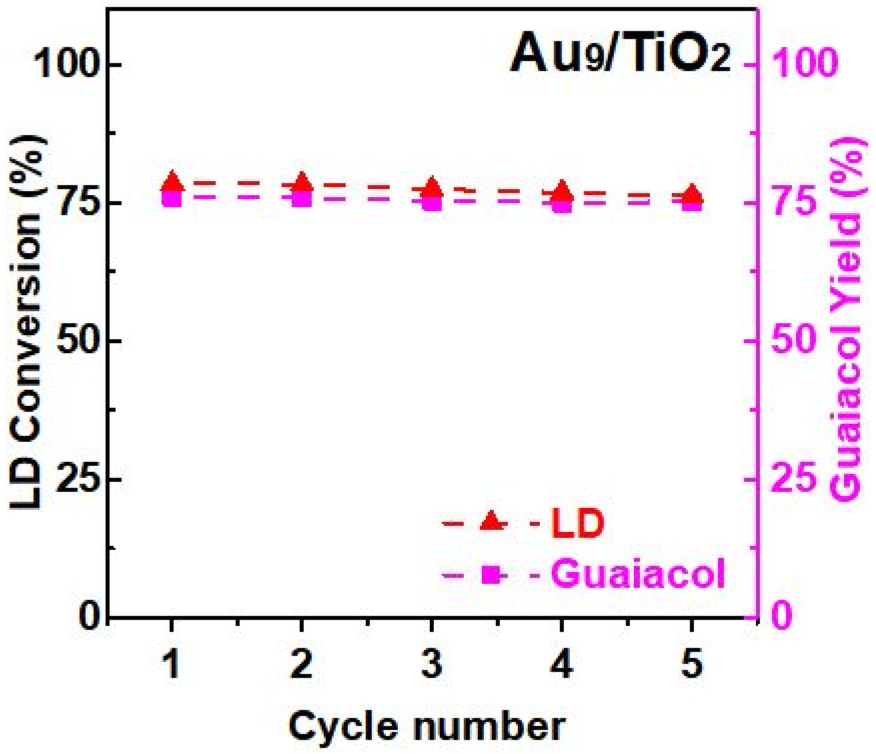

Figure S17. Durability test of Au_9/TiO_2 catalyst in the decomposition of LD.

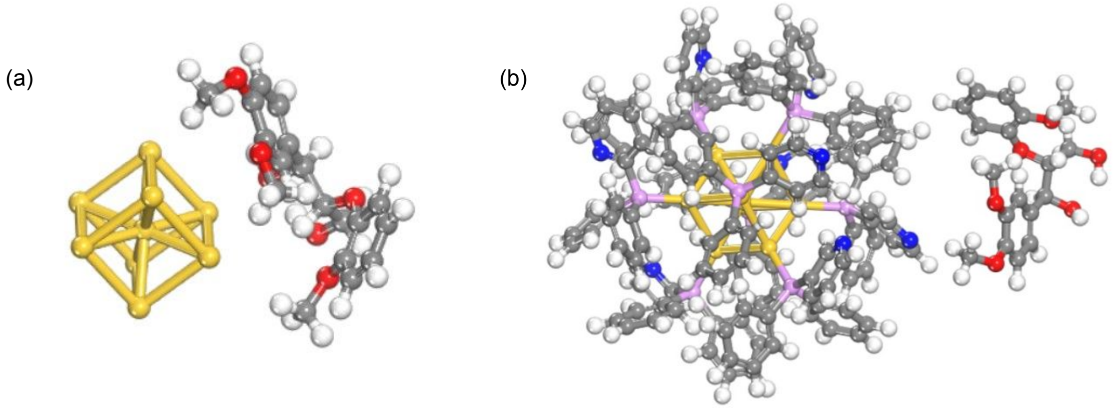

Figure S18. Lignin-cluster interaction complexes for (a) the bare Au_9 cluster and (b) the fully ligated Au_9 cluster. Interaction energies are -0.35 eV for the bare cluster and -0.95 eV for the fully ligated cluster.

Supporting Tables

Table S1. Crystal data and structure refinement for cubic $Au_9(Dppy)_8$ cluster.

Identification code	
Empirical formula	$[Au_9P_8C_{136}N_8H_{112}](SbF_6)_3$
Formula weight	4586.04
Temperature/K	173.06
Crystal system	cubic
Space group	*Pn-3n*
a/Å	18.99320(10)
b/Å	18.99320(10)
c/Å	18.99320(10)
α/°	90
β/°	90
γ/°	90
Volume/Å^3	6851.64(11)
Z	2
Radiation	CuKα (λ = 1.54178)
2θ range for data collection/°	6.582 to 132.868
Reflections collected	48075
Independent reflections	1011 [R_{int} = 0.0526, R_{sigma} = 0.0207]
Data/restraints/parameters	1011/39/84
Goodness-of-fit on F^2	1.313
Final R indexes [I>=2σ (I)]	R_1 = 0.1028, wR_2 = 0.2372
Final R indexes [all data]	R_1 = 0.1042, wR_2 = 0.2380

Table S2. Catalyst performance in the depolymerization of lignin dimer (LD).

Entry	Catalyst	Guaiacol (%)		3,4-dimethoxytoluene		LD conversion (%)
		Yield	Selectivity	Yield	Selectivity	
1	TiO_2	12.36	82.5	13.15	87.8	14.98
2	$[Au_9(Dppy)_8]^{3+}$	-	-	-	-	2.78
3	$[Au_9(Dppy)_8]^{3+}/TiO_2$	92.03	99.6	59.66	64.6	92.42
4	$Au_9(C)/TiO_2$	65.00	96.7	41.56	61.9	67.20
5	Au_{11}/TiO_2	57.89	97.3	39.80	66.9	59.50
6	Au_{13}/TiO_2	46.73	96.8	31.59	66.8	47.3
7	Au_{25}/TiO_2	51.31	96.1	35.99	67.4	53.4
8	$Au_{13}Ag_{12}/TiO_2$	68.26	94.7	63.55	88.2	72.07
9	Au NPs/TiO_2	35.62	94.4	31.49	83.4	37.75
10	Au_9/TiO_2	76.01	96.8	63.45	80.8	78.52
11	Cycle 2	75.91	96.9	59.10	75.4	78.36
12	Cycle 3	75.41	97.3	57.65	74.4	77.48
13	Cycle 4	75.06	97.6	58.53	76.1	76.87
14	Cycle 5	75.20	98.6	58.35	76.5	76.27

Reaction conditions:

100 mg LD, 5 mg catalyst, 10 mL THF, H_2 (4 MPa), 400 ^oC, 12 h. The conversion of LD was determined by GO-FID using pure products as standards.

References

1 Bosch-Navarro, C. *et al.* Covalently Binding Atomically Designed Au_9 Clusters to Chemically Modified Graphene. *Angew Chem Int Ed Engl* **54**, 9560-9563, doi:10.1002/anie.201504334 (2015).

2 McKenzie, L. C., Zaikova, T. O. & Hutchison, J. E. Structurally similar triphenylphosphine-stabilized undecagolds, $Au_{11}(PPh_3)_7Cl_3$ and $[Au_{11}(PPh_3)_8Cl_2]Cl$, exhibit distinct ligand exchange pathways with glutathione. *Journal of the American Chemical Society* **136**, 13426-13435, doi:10.1021/ja5075689 (2014).

3 Qin, Z. *et al.* Atomically precise nanoclusters with reversible isomeric transformation for rotary nanomotors. *Nature Communications* **11**, 6019, doi:10.1038/s41467-020-19789-4 (2020).
